# Integrated Care for Multimorbidity Population in Asian Countries: A Scoping Review

**DOI:** 10.5334/ijic.6009

**Published:** 2022-03-16

**Authors:** Jiaer Lin, Kamrul Islam, Stephen Leeder, Zhaohua Huo, Chi Tim Hung, Eng Kiong Yeoh, James Gillespie, Hengjin Dong, Jan Erik Askildsen, Dan Liu, Qi Cao, Benjamin Hon Kei Yip, Adriana Castelli

**Affiliations:** 1 Jockey Club School of Public Health and Primary Care, Chinese University of Hong Kong, Hong Kong SAR, China; 2 Health Services and Health Economics, NORCE Norwegian Research Centre, Bergen, Norway; 3 Department of Economics, University of Bergen, Norway; 4 Menzies Centre for Health Policy and Economics, Sydney School of Public Health, The University of Sydney, Australia; 5 Centre for Health Systems and Policy Research, Jockey Club School of Public Health and Primary Care, Chinese University of Hong Kong, Hong Kong SAR, China; 6 School of Medicine, Zhejiang University, China; 7 Centre for Health Economics Research and Evaluation, University of Technology Sydney, Australia; 8 School of Public Administration and Policy, Renmin University of China, China; 9 Centre for Health Economics, University of York, UK

**Keywords:** integrated care, multimorbidity, chronic, Asia, scoping review

## Abstract

**Background:**

The complex needs of patients with multiple chronic diseases call for integrated care (IC). This scoping review examines several published Asian IC programmes and their relevant components and elements in managing multimorbidity patients.

**Method:**

A scoping review was conducted by searching electronic databases encompassing Medline, Embase, Scopus, and Web of Science. Three key concepts – 1) integrated care, 2) multimorbidity, and 3) Asian countries – were used to define searching strategies. Studies were included if an IC programme in Asia for multimorbidity was described or evaluated. Data extraction for IC components and elements was carried out by adopting the SELFIE framework.

**Results:**

This review yielded 1,112 articles, of which 156 remained after the title and abstract screening and 27 studies after the full-text screening – with 23 IC programmes identified from seven Asian countries. The top 5 mentioned IC components were service delivery (n = 23), workforce (n = 23), leadership and governance (n = 23), monitoring (n = 15), and environment (n = 14); whist financing (n = 9) was least mentioned. Compared to EU/US countries, technology and medical products (Asia: 40%, EU/US: 43%-100%) and multidisciplinary teams (Asia: 26%, EU/US: 50%–81%) were reported less in Asia. Most programmes involved more micro-level elements that coordinate services at the individual level (n = 20) than meso- and macro-level elements, and programmes generally incorporated horizontal and vertical integration (n = 14).

**Conclusion:**

In the IC programmes for patients with multimorbidity in Asia, service delivery, leadership, and workforce were most frequently mentioned, while the financing component was least mentioned. There appears to be considerable scope for development.

**Highlights:**

- First scoping review to synthesise the key components and elements of integrated care programmes for multimorbidity in Asia.
- All programmes emphasized ‘distinctive service delivery’, ‘leadership’, and ‘workforce’ components.
- ‘Financing’ component was least mentioned in identified integrated care programmes.

## Introduction

Multimorbidity refers to the co-occurrence of more than one chronic health condition in the same individual [1]. Compared to patients with a single disease, patients with multimorbidity often have lower levels of health and health-related quality of life [2], poorer functional status [3], and a higher mortality rate [4]. If physicians follow single disease-based clinical guidelines [5,6], it will result in duplication of unnecessary services for patients with multiple problems [7,8]. This phenomenon is still commonly seen in many healthcare systems, where current clinical management approaches are mostly guideline-driven [6] and focused on patients having a single disease [9,10,11].

The complex needs of patients with multiple chronic diseases call for integrated care [12], which brings a broad range of professionals together to coordinate services. The World Health Organisation (WHO) defines integrated care (IC) as “the management and delivery of health services so that people receive a continuum of health promotion, disease prevention, diagnosis, treatment, disease-management, rehabilitation, and palliative care services, according to their needs over time that is coordinated across different levels and sites of care within the healthcare system” [13].

Integrated care can be analysed in several ways. The Rainbow Model (RMIC) developed by Valentijn et al. [14] distinguishes between three levels of integration. Constructs of integration are assigned to the three levels and the two dimensions. At the macro-level, integrated care happens at a system-level, through policy and regulatory frameworks. At the meso-level, it is described as professional integration and organisational integration through interdisciplinary collaboration, shared accountability, and inter-organisational strategy. At the micro-level, clinical integration is achieved, which refers to joint case management or care plan. Support functions and activities such as information management or financing system and shared mission or values were proposed as mechanisms to link the three levels of integration, denoted as functional integration and normative integration, respectively. RMIC also defined the integration strategies according to vertical and horizontal dimensions. Vertical integration refers to care across different settings by connecting community and hospital-based professionals [15]. By contrast, horizontal integration involves care from a person-centred perspective to improve overall health, using peer-based and cross-sectoral collaboration in the same care setting [15].

Wagner’s Chronic Care Model (CCM) [16] and Guided Care Model (GCM) [17] are two further IC models that were most often referred to when studying patients with multimorbidity [18]. The CCM comprises six domains: community; health system; self-management support; delivery system design; decision support; and clinical information system. While the GCM builds upon CCM as a proactive care model for people with multimorbidity. It contains eight elements: care assessment; planning; monitoring; coaching; chronic disease self-management; educating and supporting caregivers; coordinating transitions between providers and care sites; and access to community services. Other IC models such as The Development Model of Integrated Care [19], WHO Innovative Care for Chronic Conditions Model [20], 10-step Integrated Care Framework [21] was also developed and applied in the design and operation of IC programmes.

Initial results from evaluations show IC leads to improved patient satisfaction [22,23,24] and reductions in mortality [7], unnecessary hospital utilisation [25] and costs [26]. However, there is a lack of agreement on the fundamental components of IC programmes for patients with multimorbidity. Threapleton et al. [27] reported care continuity; enabling governance; and shared values as essential components. Poitras et al. [28] recently identified decision-process supports; patient-centred provision; and self-management, amongst others, as the core components. Struckmann et al. [18] reported that alongside comprehensive assessment and the multidisciplinary team, self-management; person-centred care; and an electronic information system were valuable; while Briggs et al. [29] found that case management was also critical. The framework adopted in these reviews to synthesise IC components varies greatly. The identified programmes included were in different contexts, mainly from Europe (including the UK, Sweden, Netherlands, Switzerland, Spain, Germany, Italy, and France) or the US [30,31,32].

In Asia, healthcare expenditure has increased substantially with socio-economic growth and development. Nonetheless, in 2020, total spending in health care as a share of GDP was only 4.7% on average compared to 8.7% in OECD countries, with private expenditure accounting for 47.4%, mainly in the form of out-of-pocket payment [33]. High levels of out-of-pocket payment cause financial barriers to access care, especially for the vulnerable population such as patients with multimorbidity. To protect against catastrophic expenditure, Japan, South Korea, Thailand, Taiwan, and China established social health insurance systems. However, public health expenditure is predominantly financed by taxation, where public healthcare resources are inadequately allocated, and some care is more reliant on the private sector, such as primary care [34]. This poses great challenges in integrating care across public and private sectors and coordinating primary, secondary, and tertiary care. The challenge is even more daunting in the context of the rapid rise in ageing in Asia. As multimorbidity increases with age [35], the vast demand for IC causes a greater burden to the existing health and social care systems. To our knowledge, no reviews have reported on IC programmes in Asia for patients with multimorbidity with a specific focus on what components are commonly applied in these programmes.

To bridge the knowledge gap, the primary objective of this scoping review was to identify existing IC programmes for the multimorbidity populations in Asian countries. Second, we aimed to summarise the most and least frequently used IC components and elements from current IC programmes. Finally, we summarised and discussed the differences between our findings on IC components and those highlighted in previous reviews in western countries.

## Methods

### Study design

Scoping reviews seek to rapidly map key concepts in a broad or complex area that have not been comprehensively reviewed in recent times. We followed the methodology of Armstrong et al. [36] and PRISMA (Preferred Reporting Items for Systematic Reviews and Meta-Analyses) guidelines [37] to conduct this review. Our scoping study aimed to cover all available materials to descriptively identify components and elements of IC programmes, rather than synthesising effective programmes or interventions. So we did not attempt to assess the quality of included studies to determine the quality of evidence, and diverse study designs were all included [38].

### Search strategy

We first appraised recent reviews [7,12,18,27,29,32,39,40] published between 2014 and 2019 to identify relevant studies on our research topic. We compiled a list of terms and Medical Subject Headings (MeSH) synonyms and grouped them under the following key concepts of interest:

1. integrated care;
2. multimorbidity;
3. asian country.

Further details are provided in Table S1 (see Appendix 1).

Search strategies combined research concepts, including title, abstract, keywords, MeSH terms, or subjects where applicable (see Appendix 2). Electronic databases including Medline, Embase, Scopus, and Web of Science were searched for relevant literature published from 1990 to 18^th^ June 2020. We then screened the bibliographies of included studies and relevant reviews for other potential articles. Third, several key journals related to integrated care were hand searched, including the International Journal of Integrated Care, Journal of Integrated Care, International Journal of Care Coordination, to capture studies not found in the initial search in the databases. Last, networks of organisations and researchers interested in IC were contacted to retrieve grey literature.

### Study selection

Studies conducted in any Asian country were included if an IC programme for persons with multimorbidity was described or about to be evaluated. Eligibility was also extended to frail patients, those with chronic diseases, or a high risk of readmission requiring the same complex care as patients with multimorbidity. Since older persons are more likely to suffer from multiple chronic conditions [41], with multimorbidity prevalence ranging between 55% to 98% [42,43], we also included IC programmes targeting older people. Articles were excluded if they 1) focused on single diseases; 2) were not related to integrated care; and 3) were conference abstracts, letters to the editor, editorials, or commentaries; 4) the target population was exclusively <18 years, patients with AIDS or addiction; 5) focused on long-term care systems or end-of-life care; 6) or were not carried out in Asian countries. Only studies published in English were included.

Two independent reviewers (JL, ZH) screened the titles and abstracts for relevance after removing duplicates. Full texts of publications were then retrieved and read independently by the same two reviewers (JL, ZH). Studies were included if both reviewers (JL, ZH) reached a consensus. The two reviewers discussed any disagreements first and then referred to the project team for consensus, if necessary.

### Data extraction and synthesis

We designed, piloted, and revised data collection forms to extract information from the included studies, including first author/year; paper title; country/region; study aims; study design; participant characteristics; care settings; descriptions of the intervention and (or) control; and summary of results. Owing to the heterogeneity of study designs, interventions, and outcome measurements, we only synthesised outcomes and recorded them as positive, negative, or insignificant. Elements and components reported in the identified IC programmes were classified and summarised using the SELFIE framework (Sustainable intEgrated care modeLs for multimorbidity delivery, FInancing and performance) [44].

The SELFIE conceptual framework for integrated care for patients with multimorbidity was recently developed from a scoping review and international expert meetings to aid the development, implementation, description, and evaluation of IC for the multimorbidity population [44]. “SELFIE” groups a wide range of IC concepts into six IC components based on the WHO six building blocks of the health system [45]: service delivery; leadership and governance; workforce; financing; technologies and medical products; and information and research, with each component examined at three levels: macro, meso, and micro. The core of the framework is a holistic understanding of the individual with multimorbidity in their environment. The triple aims (improving care experience and population health, and reducing costs) [46] were monitored across all components, noted as “monitoring functioning” [44]. The comprehensive inventory of six integrative care components emphasises integrated care as a process that spans different domains and levels. We described IC programmes with a list of IC components and elements from the SELFIE framework and synthesised the research findings for patients with multimorbidity in Asian countries.

Furthermore, horizontal or vertical integration [14] was indicated by whether care providers involved were in the same or different care setting (primary, secondary, or tertiary care settings), respectively. One reviewer (JL) conducted the extraction work, which was subsequently checked by a second reviewer (ZH).

## Results

### Characteristics of included studies

**Figure 1:**
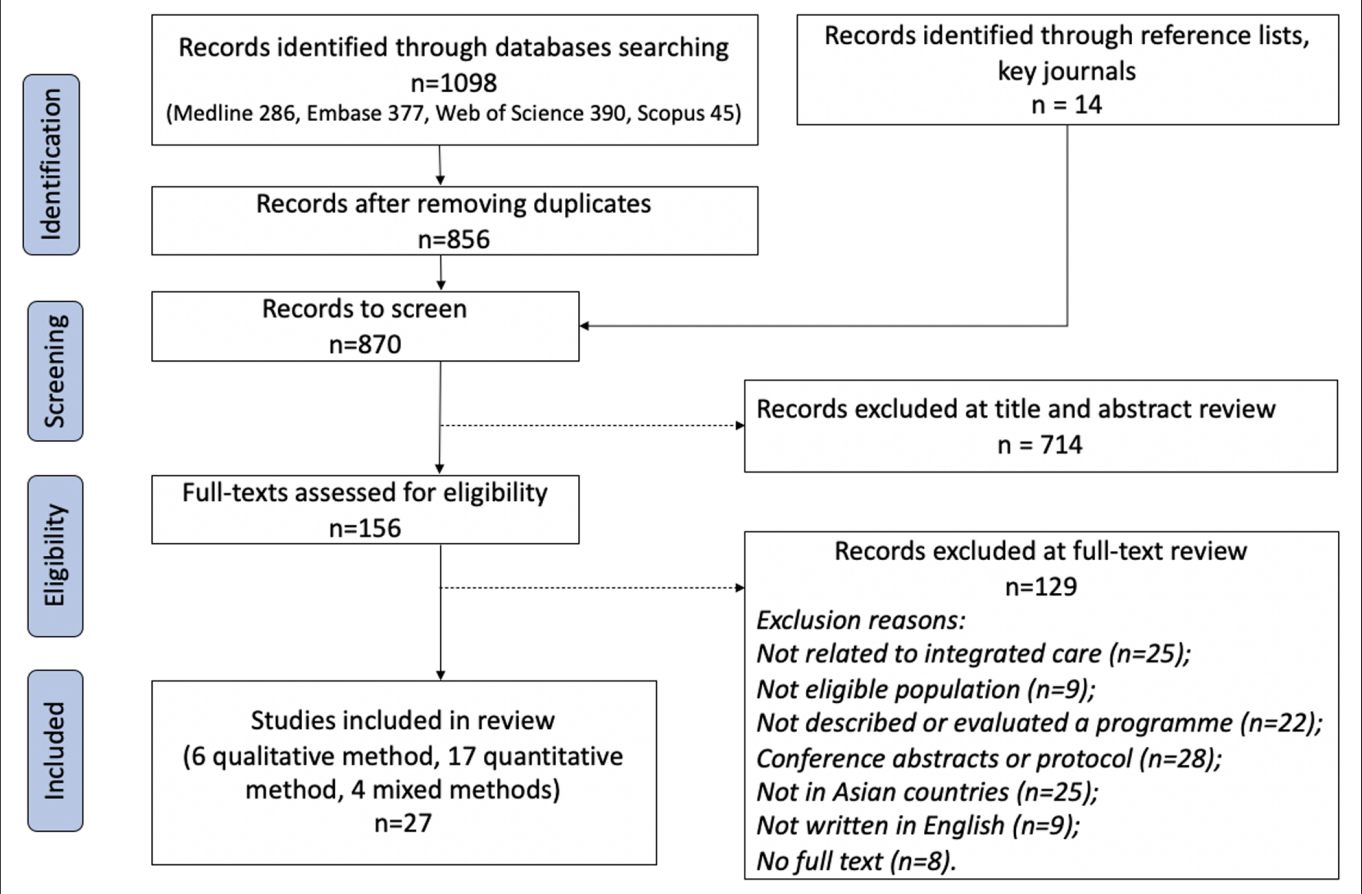
PRISMA flow diagram.

The search strategies yielded 1,098 articles from databases and 14 from searching reference lists and key journals. 870 articles remained after screening titles and abstracts for duplicates, of which 156 were considered potentially relevant and underwent full-text screening for inclusion. Finally, 27 studies were included in this review. ***Figure 1***. illustrates the study selection process. See the abbreviated list for names of IC programmes in Table S2 (see Appendix 3) and a summary of the studies reviewed in Table S3 (see Appendix 4).

Among the 27 included articles, nine studies were conducted in Singapore [47,48,49,50,51,52,53,54,55], followed by seven in Hong Kong SAR [56,57,58,59,60,61,62], four in mainland China [63,64,65,66], three in Japan [67,68,69], two in India [70,71], one in Taiwan [72], and one in Thailand [73] – all published between 1999 and 2020, with half published since 2018. ***Table 1*** summarises the characteristics of included studies and IC programmes.

The most common study design used quantitative methods (63%, n = 17), including seven randomised control trials, four retrospective cohort studies, two quasi-experimental studies, and four others. Six of 27 (22.2%) studies used qualitative methods, and the remaining four articles (14.8%) were mixed-method studies.

**Table 1:** Summary of characteristics of included studies and IC programmes.


	NO. OF STUDIES	REFERENCE

**Study design**		

*Qualitative*	**6**	

Descriptive study	4	[51,70,66,54]

Interviews and focus group discussions	2	[63,64]

*Quantitative*	**17**	

Retrospective cohort study	4	[53,48,62,69]

Randomised controlled trial	7	[58,61,57,56,60,71]

Quasi-experimental study	2	[47,49]

Retrospective longitudinal study	1	[68]

Longitudinal and quasi-experimental study	1	[65]

Difference-in-difference design	1	[72]

Cross-sectional survey	1	[73]

*Mixed methods*	**4**	[67,59,50,55]

**Reported quantitative outcome**		

Increase activities of daily life (ADL)	1	[67]

Increase health-related quality of life (HRQoL)/health utility	3	[57,56,53]

Increase self-rated health	2	[60,53]

Increase self-efficacy	2	[57,60]

Increase patient satisfaction	2	[57,52]

Reduce length of stay (LOS)^a^	2	[61,58]

Reduce hospital admissions	5	[61,58,49,48,62]

Reduce hospital readmission rate/30-day readmission^b^	5	[56,57,60,53,48]

Reduce outpatient attendances^c^	1	[58]

Reduce emergency departments attendances^d^	3	[53,49,48]

Reduce cost	3	[58,56,65]

Reduced Continuity of Care Index (COCI)	1	[72]

Low or moderate implementation fidelity	1	[50]

Increase cost	1	[47]

	**NO. OF PROGRAMMES**	**NAME OF PROGRAMMES**

**Care setting(s)**		

Inpatient setting	1	Acute Medical Unit [51]

Community setting	3	Integrated Community Care System [68], Training Programmes for Primary Care Teams [73], mWellcare Programme – mHealth-based electronic decision support system [70,71]

Home setting	5	home healthcare services provided with videophones [67], Health-Social Transitional Care Management Program [56,57], Nurse-led Case Management Programme [60], Singapore General Hospital Transitional Home Care Programme [49], Transitional Home Care-Integrated Practice Units Programme [48]

Inpatient + ambulatory setting	1	The Reform of the Integrated County Healthcare Consortium [65]

Inpatient + ambulatory + community setting	1	Joint Health Centre pilot programme [63]

Inpatient + community setting	2	emergency physician–led frailty-care unit [62], discharge services [69]

Inpatient + community + home setting	1	Regional Health System [54]

Inpatient + home setting	3	The Aged Care Transition Program [53], National University Health System-Regional Health System Transitional Care Programme [50], National University Health System-Regional Health System Integrated Interventions and Care Extension programme [47]

Ambulatory + community setting	3	The Reform of Integrated Service Delivery Network-Luohu Hospital Group[64,66], Family Doctor Plan [72], Right-Site Care program [55]

Ambulatory + home setting	1	Singapore General Hospital Transitional Care Programme [52]

Ambulatory + community + home setting	1	Targeted Case Management [58,59]

Community + home setting	1	Case Management [61]

**Target population**		

Elderly	5	Home Healthcare Services Provided with Videophones [67], Integrated Community Care System [68], Health-Social Transitional Care Management Program [56,57], Hospital Discharge Services [69], Emergency Physician–led Frailty-care Unit [62]

Frail elderly	2	Case Management [61], Transitional Home Care-Integrated Practice Units Programme [48]

Multimorbidity	8	Targeted Case Management [58,59], Taiwan’s Family Doctor Plan [72], Training Programmes for Primary Care Teams [73], The Aged Care Transition Program [53], Singapore General Hospital Transitional Home Care Programme [49], Nurse-led Case Management Programme [60], National University Health System-Regional Health System Transitional Care Programme [50], mWellcare Programme – mHealth-based electronic decision support system [70,71]

Patients with a high risk of readmission	2	Singapore General Hospital Transitional Care Programme [52], National University Health System-Regional Health System Integrated Interventions and Care Extension programme [47]

Patients with chronic diseases	3	Joint Health Centre for chronic care [63], Right-Site Care program [55], The Reform of Integrated Service Delivery Network-Luohu Hospital Group [64,66]

N/R	3	Regional Health System [54], Acute Medical Unit [51], The Reform of the Integrated County Healthcare Consortium [65]


^a^ One article [49] reported a non-significant change in length of stay (LOS). ^b^ Three articles [52,62,69] reported non-significant changes in hospital readmission rate. ^c^ One article [48] reported a non-significant change in outpatient attendances. ^d^ One article [52] reported a non-significant change in emergency departments attendances.

Concerning the effectiveness of IC programmes, most studies (64%, n = 16) reported healthcare utilisation outcomes such as hospital (re)admission (n = 13), outpatient or emergency department attendances (n = 6), and length of stay (n = 3). By contrast, patient-centred outcomes (i.e., quality of life, patient satisfaction, self-rated health, self-efficacy, and activities of daily living) contributed less (36%, n = 9). Nearly all studies reported positive outcomes of IC programmes except for one where the outcome was the continuity of care index and another where implementation fidelity was defined as the outcome.

### Characteristics of included IC programmes

In general, 60% of articles (n = 14) reviewed included programmes covering more than one care setting. Only two of 23 IC programmes were proposed following existing IC models. For example, China’s “Luohu Group” was designed based on the RMIC model [74] and Singapore’s Right-Site Care (RHC) programme was developed following the Patient-Centred Medical Home Model [75] and the Primary Care Plus Model [76]. Eight of the IC programmes targeted multimorbidity (34.8%), five for the elderly (21.7%), followed by patients with chronic diseases (n = 3, 13%) and those with a high risk of readmission (n = 2, 8.7%). ***Table 2*** provides a description of IC programmes. For care providers, most programmes involved nurses (n = 15, 65.2%), followed by physicians (n = 13, 56.5%) and social workers (n = 11, 47.9%). Only six programmes (26.1%) emphasised the role of multidisciplinary teams and four (17.4%) of allied health professionals.

**Table 2:** Description of integrated care programmes in the included studies.


PROGRAMME NAME	PAPER TITLE	COUNTRY/REGION	PROGRAMME DESCRIPTION	POLITICAL BACKGROUND IN THE LOCAL CONTEXT	PROVIDER(S) INVOLVED

Home Healthcare Services Provided with Videophones (HHSPV programme, launched in 1991)	The effectiveness of videophones in home healthcare for the elderly [67].	Japan	The programme provided medical consultation; comprehensive assessments; instructions for patients and healthcare workers; advice on the effective use of health and welfare resources; and emotional support for caregivers	The growth of the ageing population is rapid in Japan, and there are great demands on more professionals involved and coordinated services in homecare. Telecommunications between households and healthcare providers became available to meet the needs of the elderly.	physician; nurse; allied health professionals; social worker; home helpers

Case Management (CM programme, launched in 2000)	Cost-benefit analysis of a case management project for the community-dwelling frail elderly in Hong Kong [61].	Hong Kong	The programme provided home visit and telephone follow-up; comprehensive geriatric assessment; care plan; coordination between health and social services; monthly monitoring via IT system; health and psychosocial counselling and health educational programmes	N/A	social worker; registered nurse (case manager); an interdisciplinary team

Targeted Case Management (TCM programme, launched in 2001)	Reducing utilisation of hospital services by case management: a randomised controlled trial [58]. Global case management: Hong Kong. Care for the hospital-discharged frail elders by nurse case managers: a process evaluation of a longitudinal case management service project [59].	Hong Kong	The programme provided regular monitoring of subjects’ health status; daily phone assistance; home visits; community-based supportive services; access to medical support	Hong Kong is experiencing a growing ageing population. For post-hospital discharged elderly, access to different services is all the more daunting, given fragmented medical and social service structures and the disparate health and social care financing arrangements in Hong Kong.	nurse case managers; case geriatricians; a multidisciplinary team

Taiwan’s Family Doctor Plan (FDP programme, launched in 2006)	Impact of integrated healthcare: Taiwan’s Family Doctor Plan [72].	Taiwan	The programme provided multidisciplinary teams; case management; integrated care pathways	The care delivery system in Taiwan lacks features or context of strong primary care. Taiwanese patients do not have family doctors and have access to hospital care without a referral. FDP pays office-based physicians under a national contract to provide pre-specified elements of integrated care to patients assigned to them.	physicians

Training Programmes for Primary Care Teams (TPPCT programme, launched in 2006)	Assessing system-based trainings for primary care teams and quality-of-life of patients with multimorbidity in Thailand: patient and provider surveys [73].	Thailand	The programme provided integrated primary care systems management, including resource sharing; community participation and inter-sectoral collaboration; health information systems; management skills of the leaders; coordination and unity of teamwork; integrated service delivery	In Thailand, primary healthcare has long focused on national healthcare reforms and adopted a “task-shifting” strategy as a long-term approach to training health workers to work in primary care settings. Since 2006, a series of 11 training programmes for multidisciplinary primary care teams have been implemented nationwide.	physicians; a multidisciplinary team

The Aged Care Transition Program (ACTION programme, launched in 2008)	Effectiveness of a National Transitional Care Program in Reducing Acute Care Use [53].	Singapore	The programme provided comprehensive assessment; medical services; social services; self-management support; care planning; follow-up through calls and home visits; coordination of referral	Singapore’s community and long-term care systems are less well developed than its acute care system. Care integration and enabling better and more-comprehensive chronic and long-term care in the nonacute sector is a priority for the Health Ministry. This gap results in a vicious cycle of discharge and readmissions to acute care hospitals. Thus, transitional care support is needed.	physicians; care coordinators; project director

Singapore General Hospital transitional home care programme (SGH-THC programme, launched in 2008)	Effectiveness of a transitional home care program in reducing acute hospital utilization: a quasi-experimental study [49].	Singapore	The programme provided comprehensive medical and nursing assessment including optimising medical conditions in the home setting; education on self-management; reducing polypharmacy and medication conflicts; facilitating adherence to treatment; ensuring follow up by specialists; activating appropriate community services; care planning; telephone call reviews; early physician reviews	Singapore’s community and long-term care systems are less well developed than its acute care system. Care integration and enabling better and more-comprehensive chronic and long-term care in the nonacute sector is a priority for the Health Ministry. This gap results in a vicious cycle of discharge and readmissions to acute care hospitals. Thus, transitional care support is needed.	family physician; nurse case manager; physiotherapist; occupational therapist; speech therapist; medical social worker

Integrated Community Care System (ICCS programme, launched in 2009)	“Ageing in Place” Policy in Japan: Association Between the Development of an Integrated Community Care System and the Number of Nursing Home Placements Under the Public Long-Term Care Insurance Program Among Municipal Governments [68].	Japan	The programme provided prevention of long-term care; support for case managers; case conference; discharge planning; home healthcare; end of life care at home; housing provision; improved home environment; prevention of social isolation; suicide prevention; disaster risk management; advocacy for elderly; elder abuse prevention and intervention	In 2000, a national long-term care insurance (LTCI) programme for the elderly was introduced to differentiate social care from healthcare and to help people age in place. In 2006, community-based care services and community general support centres in municipalities were introduced into the LTCI programme to establish one-stop home care services. The national government published a model of the “Integrated Community Care” system model.	care manager; home care service provider; community-based service provider; residential care service provider; housing service provider

Nurse-led Case Management Programme (NCM programme, launched in 2010)	A randomized controlled trial of a nurse-led case management programme for hospital-discharged older adults with co-morbidities [60].	Hong Kong	The programme provided comprehensive pre-discharge needs assessment; development of goal and care plan with patients; analysis of performance barriers with patients; monitoring of system; home visit for post-discharge assessment; training of nursing students; support in maintaining self-management behaviours	N/A	nurse case managers; nursing students

Health-Social Transitional Care Management Program (HSTCM programme)	Effects of a health-social partnership transitional program on hospital readmission: A randomized controlled trial [57]. Cost-effectiveness of a health-social partnership transitional program for post-discharge medical patients [56].	Hong Kong	The programme provided home visits; assessment in the holistic domains; relevant intervention; social support; follow-up telephone calls; social assessment and interventions; regular case reviews	N/A	nurse case manager; trained volunteers; social workers

Singapore General Hospital Transitional Care Programme (SGH-TC programme launched in 2011)	Transitional care for the highest risk patients: findings of a randomised control study [52].	Singapore	The programme provided comprehensive assessment; post-discharge surveillance of the patient to ensure adherence to care plans; coordination of follow-up visits with specialist care providers; patient education and caregiver coaching; activation of community and social services	Singapore’s community and long-term care systems are less well developed than its acute care system. Care integration and enabling better and more-comprehensive chronic and long-term care in the nonacute sector is a priority for the Health Ministry. This gap results in a vicious cycle of discharge and readmissions to acute care hospitals. Thus, transitional care support is needed.	doctor; advance practice nurse; nurses; medical social worker; physical therapists; pharmacists

Regional Health System (RHS programme, launched in 2012)	Implementation of Integrated Care in Singapore: A Complex Adaptive System Perspective [50].	Singapore	The programme provided a network led by a major public hospital, close partnership with other healthcare providers; social care providers within the same geographical region	As the demands for healthcare services increase with ageing, Singapore realised that a disease-centred provision of services within the hospitals is insufficient and unsustainable in the long term. The RHS model was introduced by the Ministry of Health in 2012 to foster integrated care within respective geographic regions.	major public hospital; primary care providers; community hospitals; nursing homes; home care; day rehabilitation providers; social care providers

National University Health System-Regional Health System Transitional Care Programme (NUHS-RHS TC programme, launched in 2012)	Implementation fidelity of a strategy to integrate service delivery: learnings from a transitional care program for individuals with complex needs in Singapore [47]. Retrospective evaluation of healthcare utilisation and mortality of two post-discharge care programmes in Singapore [54].	Singapore	The programme provided needs assessments; development of personalised care plans; care coordination; case closure	The RHS in Singapore was designed to integrate hospitals with primary and community care partners within a geographical region. The National University Health System (NUHS) RHS is one of the RHS systems in Singapore and launched two programmes to improve post-discharge care and anchor care in the community and reduce healthcare utilisation in 2014: (1) NUHS-RHS Integrated Interventions and Care Extension (NICE) programme and (2) NUHS Transitional Care Programme (NUHS TCP).	care coordinators; multidisciplinary care team; medical care providers; social care workers

Joint Health Centre for chronic care (JHC programme, launched in 2013)	Integrated care reform in urban China: a qualitative study on design, supporting environment and implementation [63].	China	The programme provided integrated care of primary health care and specialist; care in coordinated hospitals; referral to different levels of care facilities; follow-up by GPs	China initiated some pilot integrated care models between hospitals and community health centres (CHCs) in Henan Province, Qinghai Province, to develop a vertical referral system among health providers. As one of the pilots, the Hangzhou municipal government implemented some innovations. The integrated care model was created, namely the Joint Health Centre (JHC) for chronic care.	general practitioners; nurses; specialists (hospitals)

Hospital Discharge Services (HDS programme, launched in 2013)	Associations of Hospital Discharge Services with Potentially Avoidable Readmissions Within 30 Days Among Older Adults After Rehabilitation in Acute Care Hospitals in Tokyo, Japan [69].	Japan	The programme provided discharge planning; rehabilitation discharge instruction on self-management and community care at discharge; coordination with long-term care	Japanese hospitals do not offer comprehensive transitional care programmes under the health insurance system. Some individual services such as discharge planning were covered by insurance and used a diagnosis procedure combination (DPC) system to enable case-mix adjustments for reimbursements.	nurses; medical social workers; doctors; allied health professionals; care manager

National University Health System-Regional Health System Integrated Interventions and Care Extension (NUHS-RHS NICE programme, launched in 2014)	Retrospective evaluation of healthcare utilisation and mortality of two post-discharge care programmes in Singapore [47].	Singapore	The programme provided case management for patients and caregivers; follow-up home visits and telephone calls; community-based care management	The RHS in Singapore was designed to integrate hospitals with primary and community care partners within a geographical region. The National University Health System (NUHS) RHS is one of the RHS systems in Singapore and launched two programmes to improve post-discharge care and anchor care in the community and reduce healthcare utilisation in 2014: (1) NUHS-RHS Integrated Interventions and Care Extension (NICE) programme and (2) NUHS Transitional Care Programme (NUHS TCP).	case managers; social workers; volunteers

Right-Site Care (RSC program, launched in 2014)	Shifting care from hospital to community, a strategy to integrate care in Singapore: process evaluation of implementation fidelity [55].	Singapore	The programme provided referral of suitable patients by specialists; coordination of care under one dedicated physician; stratification of care and care location; preparation of transition; support for primary and community care providers; multidisciplinary case conference to refine care plans	In 2013, within the NUHS RHS in Singapore, over 600,000 unique attendances at the SOCs in its primary acute hospital, rising from 500,000 in 2009. In response to this rapid increase in SOC utilisation, NUHS RHS imitated the Right-Site Care (RSC) programme to facilitate timely discharge and support the appropriate transition from the hospital to the community.	care coordinators; family physicians; a team of healthcare workers

Acute Medical Unit (AMU programme, launched in 2015)	Acute medical unit: experience from a tertiary healthcare institution in Singapore [51].	Singapore	The programme provided early internist-led assessment and management; holistic management; active re-triaging of the patient; inpatient treatment triggered; allied health services; clinical management and services support	The care stratum of dealing with admitted patients with multiple diseases and complex conditions is not well established in the Singapore healthcare model. National University Hospital in Singapore collaborated with the emergency department to create an acute medical team and scaled it up to an acute medical unit.	internists; nurses; allied health professionals

Transitional Home Care-Integrated Practice Units Programme (THC-IPU programme, launched in 2015)	Transitional Home Care Program Utilizing the Integrated Practice Unit Concept (THC-IPU): Effectiveness in Improving Acute Hospital Utilization [48].	Singapore	The programme provided comprehensive medical and nursing assessment; reconciliation; caregiver education; personal health record; multidisciplinary team meetings; telephone follow up; allied health services; home visits; readmissions review	In Singapore, nearly a quarter of semi-ambulant and non-ambulant Singapore elderly stay alone or with their elderly spouse. At the same time, primary care and home care in Singapore are relatively less developed than tertiary care. In 2016, the Ministry of Health decided to transit it to a new transitional care model named “Hospital to Home”.	junior physicians; home care nurses; allied health professionals; pharmacist; medical social worker; administrators; senior family physicians

The Reform of Integrated Service Delivery Network-Luohu Hospital Group (Luohu Group programme, launched in 2015)	The Luohu Model: A Template for Integrated Urban Healthcare Systems in China [66]. Building a People-Centred Integrated Care Model in Urban China: A Qualitative Study of the Health Reform in Luohu [64].	China	The programme provided integrated health care with public health services and social services; merged resources of hospitals and community health centres; training providers; consolidated professional resources; a formal two-way referral system, timely decision support; shared information system; guide by a “health-centred” perspective; a shared goal	In 2016, the Chinese government proposed strengthening healthcare in China through a tiered health care delivery system following a People-Centred Integrated Care model. A guideline for constructing Medical Consortia suggested hospital groups in urban areas, medical associations in rural areas, cross-regional specialist alliances, and telecollaboration networks. In 2017, China’s National Health and Family Planning Commission introduced the Luohu model to achieve “less illness, fewer hospital admissions, lower financial burdens, and better services”.	the district government; the municipal government; hospitals; health workers in community health centres; family doctor team

The Reform of the Integrated County Healthcare Consortium (ICHC programme, launched in 2015)	Does capitation prepayment based Integrated County Healthcare Consortium affect inpatient distribution and benefits in Anhui Province, China? An interrupted time series analysis [65].	China	The programme integrated high-quality healthcare resources in the county to provide integrated and continuous healthcare for residents and established capitation prepayment of the New Rural Cooperative Medical System (NRCMS) funds.	In 2002, the Chinese government established the New Rural Cooperative Medical System (NRCMS) to provide rural residents with outpatient and inpatient reimbursement for some compliance costs. As rural residents in China can freely choose hospitals, and three-tiered healthcare hospitals have yet to achieve a good cooperation relationship, patients tend to go to high-level hospitals for improved treatment, which increases healthcare expense and places tremendous pressure on the NCRMS funds. In 2015, Anhui Province launched the reform of the Integrated County Healthcare Consortium (ICHC) with capitation prepayment of the NRCMS funds following the principle of “exceeds expenditures does not make up, the balance holds for use”.	county-level hospitals (CHs); township-level hospitals (THs); village clinics

Emergency Physician–led Frailty-care Unit (EPFU programme, launched in 2015)	The effectiveness of an emergency physician-led frailty unit for the living-alone elderly: A pilot retrospective cohort study [62].	Hong Kong	The programme provided a mandatory and complete input of electronic Patient Assessment Form; a comprehensive enquiry of social background; a mandatory bundled referral to the pre-discharge team; daily ward; management plan; a target of discharge within 72 h of admission	The Accident & Emergency Department of Queen Elizabeth Hospital in Hong Kong catered for 184,433 new case attendances in 2016/2017. To respond to this growing demand, a six-bed frailty-care unit dedicated to the community-dwelling elderly was established in 2015 within the Emergency Medicine Ward based on the British National Health Service (NHS) frailty model. It is the first of its kind in Hong Kong.	physicians; geriatricians; nurses; allied health professionals; social workers

mWellcare Programme – mHealth-based electronic decision support system (mWellcare programme, launched in 2016)	Development of mWellcare: an mHealth intervention for integrated management of hypertension and diabetes in low-resource settings [70]. Effectiveness of an mHealth-Based Electronic Decision Support System for Integrated Management of Chronic Conditions in Primary Care: The mWellcare Cluster-Randomized Controlled Trial [71].	India	The programme provided a generation of EDS recommendations for the management of diseases; store electronic health records; long-term monitoring and follow-up; reminder message service for scheduled medication adherence and follow-up visits; training	The burden of non-communicable diseases (NCDs), population ageing, severe shortage of skilled healthcare providers, and inadequately developed health systems impose huge constraints on healthcare services in India. The 1979 Alma Ata declaration endorsed strengthening primary care to improve health outcomes. Innovations using mobile phone technologies and task sharing by trained nurses can empower and facilitate the care process.	Community Health Centre physicians; NCD nurses


### Components of included IC programmes

***Table 3*** lists the IC components and dimensions identified for each programme, and IC elements are presented in Table S4 (see Appendix 5). Almost all IC programmes referred to the micro-level (clinical integration, n = 20). By contrast, meso-level (professional and organisational integration, n = 7) and macro-level (system integration, n = 6) integration were less common and only found in articles from mainland China, Singapore, Japan, and Thailand. Thirteen (56.5%) programmes used a combination of horizontal and vertical integration, while ten (43.5%) adopted only horizontal integration strategies.

**Table 3:** Dimension and components of integrated care programmes.


PROGRAMME NAME	TARGET POPULATION CARE SETTING	COUNTRY/REGION	DIMENSION OF INTEGRATED CARE						INTEGRATED CARE COMPONENTS^A^								
	
			MACRO-LEVEL	MESO LEVEL	MICRO-LEVEL	HORIZONTAL IC	VERTICAL IC		MM	ENVIRONMENT	SERVICE DELIVERY	LEADERSHIP	WORKFORCE	FINANCING	TECHNOLOGY	INFORMATION	MONITORING

HHSPV programme	elderly home setting	Japan			✓	✓				✓	✓	✓	✓		✓	✓	✓

CM programme	frail elderly home setting community setting	Hong Kong			✓	✓				✓	✓	✓	✓		✓	✓	✓

TCM programme	multimorbidity ambulatory setting community setting home setting (mainly)	Hong Kong			✓	✓	✓		✓	✓	✓	✓	✓				✓

FDP programme	multimorbidity ambulatory setting community setting	Taiwan			✓	✓	✓		✓		✓	✓	✓	✓			✓

TPPCT programme	multimorbidity community setting	Thailand			✓	✓	✓		✓		✓	✓	✓				

ACTION programme	multimorbidity inpatient setting home setting	Singapore			✓	✓	✓		✓	✓	✓	✓	✓	✓		✓	✓

SGH-THC programme	multimorbidity home setting	Singapore			✓	✓			✓	✓	✓	✓	✓	✓	✓	✓	✓

ICCS programme	elderly community setting	Japan	✓	✓	✓	✓				✓	✓	✓	✓	✓			

NCM programme	multimorbidity home setting	Hong Kong			✓	✓			✓		✓	✓	✓		✓		✓

HSTCM programme	elderly home setting	Hong Kong			✓	✓				✓	✓	✓	✓				✓

SGH-TC programme	patients with high risk of readmission ambulatory setting home setting	Singapore			✓	✓	✓			✓	✓	✓	✓				✓

NUHS-RHS TC programme	multimorbidity inpatient setting home setting	Singapore	✓	✓	✓	✓	✓		✓	✓	✓	✓	✓		✓	✓	✓

RHS programme	N/R inpatient setting community setting home setting	Singapore	✓	✓		✓	✓				✓	✓	✓	✓			✓

HC programme	patients with chronic diseases ambulatory setting inpatient setting community setting	China	✓	✓		✓	✓				✓	✓	✓		✓		

HDS programme	elderly inpatient setting community setting	Japan			✓	✓	✓			✓	✓	✓	✓	✓			

NUHS-RHS NICE programme	patients with high risk of readmission inpatient setting home setting	Singapore			✓	✓	✓		✓	✓	✓	✓	✓		✓	✓	✓

RSC program	patients with chronic diseases ambulatory setting community setting	Singapore		✓	✓	✓	✓				✓	✓	✓		✓	✓	

AMU programme	N/R inpatient setting	Singapore			✓	✓			✓		✓	✓	✓			✓	

THC-IPU programme	frail elderly home setting	Singapore			✓	✓			✓	✓	✓	✓	✓	✓	✓	✓	✓

Luohu Group programme	patients with chronic diseases ambulatory setting community setting	China	✓	✓	✓	✓	✓			✓	✓	✓	✓	✓	✓	✓	✓

ICHC programme	N/R ambulatory setting inpatient setting	China	✓	✓		✓	✓				✓	✓	✓	✓			

EPFU programme	elderly inpatient setting community setting	Hong Kong			✓	✓	✓			✓	✓	✓	✓		✓		

mWellcare programme	multimorbidity community setting	India			✓	✓			✓		✓	✓	✓		✓	✓	✓

**No. of programmes per dimension/component**			**6**	**7**	**20**	**23**	**14**		**11**	**14**	**23**	**23**	**23**	**9**	**12**	**11**	**15**


Abbreviations: MM = multimorbidity, N/R = Not reported, HHSPV = Home Healthcare Services Provided with Videophones, CM = Case Management, TCM = Targeted Case Management, FDP = Family Doctor Plan, TPPCT = Training Programmes for Primary Care Teams, ACTION = The Aged Care Transition, SGH-THC = Singapore General Hospital transitional home care, ICCS = Integrated Community Care System, NCM = Nurse-led Case Management Programme, HSTCM = Health-Social Transitional Care Management, SGH-TC = Singapore General Hospital Transitional Care, NUHS-RHS TC = National University Health System-Regional Health System Transitional Care, RHS = Regional Health System, JHC = Joint Health Centre, HDS = Hospital Discharge Services, NUHS-RHS NICE = National University Health System-Regional Health System Integrated Interventions and Care Extension, RSC = Right-Site Care, AMU = Acute Medical Unit, THC-IPU = Transitional Home Care-Integrated Practice Units, ICHC = Integrated County Healthcare Consortium, EPFU = Emergency Physician–led Frailty-care Unit. ^a^ According to the SELFIE Conceptual Framework, integrated care for multimorbidity population includes nine components: (1) multimorbidity, (2) environment, (3) service delivery, (4) leadership and governance, (5) workforce, (6) financing, (7) technology and medical products, (8) information and research, (9) monitoring. Programmes were marked if the concerned component was mentioned or reported in the included studies regardless of emphasising any level (micro/meso/macro).

All programmes highlighted the importance of service delivery; workforce; leadership; and governance concerning IC components. Components such as monitoring (65.2%, n = 15); environment (60.1%, n = 14); technology and medical product (52.2%, n = 12); multimorbidity (47.8%, n = 11); and information and research (47.8%, n = 11) were less frequently included, and financing was least mentioned (39.1%, n = 9). The following sections describe the specific elements extracted from the 23 IC programmes about IC components.

#### Holistic understanding of multimorbidity in his/her environment

Multimorbidity was addressed as a critical inclusion criterion in eligible populations since 2001 in the Targeted Case Management programme [58,59] and was frequently mentioned in different transitional care programmes (26.1%, n = 6). A holistic understanding of multimorbidity includes the role of an individual’s environment. A desirable environment for patients with multimorbidity should have community resources, financial and social supports easily available to patients [44]. Under the environmental component, community services were most frequently described (39.1%, n = 9), including community services assessment, referral coordination within the community, supportive patient groups, and instructions of community care. Five IC programmes (21.7%) specified home environment assessments such as home safety and home care equipment, especially before hospital discharge. Welfare resources or social support were provided in four IC programmes (17.4%). However, few programmes assessed patients’ financial situations (8.7%, n = 2), social networks (4.3%, n = 1), and no programmes provided transportation services.

#### Service delivery

All 23 IC programmes included the service delivery component. At the micro-level, pro-active (73.9%, n = 17), patient-centred (69.6%, n = 16) and tailored care (69.6%, n = 16) were most frequently addressed through case management or self-management. Most programmes (60.9%, n = 14) included coordination between providers during the transition to other care settings to facilitate continuity of care. Eleven programmes (47.8%) provided workshops or home visits for patients on developing self-management skills. Some programmes involved informal caregivers (30.4%, n = 7) by teaching them caregiving skills or providing emotional supports. Medication safety due to treatment interactions was less frequently addressed among the micro-level elements (17.4%, n = 4).

Overall, elements within the meso or macro level of service delivery were less frequently reported. The meso-level integration could be achieved by merging resources into one new integrated body such as a multipurpose day-care centre, frailty unit, or integrated practice unit (13%, n = 3). Another way was to build a virtual network among hospitals and community centres/hospitals nearby (17.4%, n = 4), commonly seen in top-down programmes from Singapore and mainland China. This was aligned with macro-level policies to improve service availability and accessibility by shortening travel distances from home to hospitals and community centres. Resources were combined from different care settings for patients’ convenience (17.4%, n = 4).

#### Leadership and governance

For the leadership and governance component, shared decision-making (65.2%, n = 15), individualised care planning (65.2%, n = 15), and coordination tailored to complexity (69.6%, n = 16) were used frequently in IC programmes. Different healthcare professionals collaborated across care settings in a multidisciplinary team to make individualised care plans together. The design of care plans also highlighted the involvement of patients. For example, the HSTCM programme [56,57] engaged patients in the case conference to set a mutual goal, increasing their treatment adherence. Several programmes used the results of comprehensive needs assessment and patients’ disease profiles at the entry of care to decide the best form of care coordination.

At the meso-level, more than half of IC programmes had supportive leadership (56.5%, n = 13), with clearly defined goals included in official documents, designed around the concept of “what is best for the patient”. Several programmes (17.4%, n = 4) shared the same organisational culture to build trust relationships through a newly merged organisation or collaborating networks. Four programmes (17.4%) clearly defined the accountability of roles (i.e., case manager, physician, and social worker). Six IC programmes (26.1%) in Singapore and mainland China adopted a performance-based assessment system for evaluating IC programmes (i.e., implementation fidelity evaluation) to ensure that the programme was on the right track. These two countries also emphasised the importance of targeting populations with chronic diseases or multimorbidity management within their policy background (34.8%, n = 8) and political commitment (30.4%, n = 7).

#### Workforce

Half of the 23 programmes assigned named coordinators or case managers, primarily nurses, to ensure continuity of care between inpatient and outpatient settings. Case managers communicated with professionals in regular case conferences on patients’ behalf, followed up and monitored care plans, and helped coordinate with social care providers. Also, a core group of professionals was found in 13 (56.5%) programmes, for example, the care transition team for ACTION programme [53], the care management team for the NICE programme [47], and the family doctor team for Luohu Group [64,66]. However, only six programmes (26.1%) mentioned multidisciplinary teams explicitly.

At the meso-level, six programmes (26.1%) offered skill-training courses for care providers. For example, the SGH-THC programme [49] provided courses for time and resource management skills. AMU [51], mWellcare [70,71] and FDP programmes [72] included training courses on multimorbidity management. Other skills like managing stress and building a trusting relationship with patients were mentioned in the TCM programme [58,59]. Informal caregivers (30.4%, n = 7) were also provided with skills training specified in the “service delivery” section. Several IC programmes (21.7%, n = 5) developed new professional roles, moving workloads from specialists to nurses or secondary care to primary care by building an internist-led unit or family doctor team. Four programmes (17.4%) responded to demographic changes and the increasing prevalence of non-communicable diseases at the macro-level. Two programmes (8.7%) designed a mentorship system to train the workforce for education and recruitment purposes.

#### Financing

Financing was the least frequently found component (39.1%), mentioned by only nine studies. At the micro-level, five IC programmes (21.7%) specified coverage or reimbursement for patients with multimorbidity. Two programmes were subsidised directly (8.7%,) and three were paid through current insurance systems. In two programmes (8.7%), patients were required to co-pay according to the complexity of their care needs. Provider-level financial incentives were addressed in only two programmes – the Luohu Group initiative [64,66] and the FDP programme [72] – by increasing funding, subsidising facilities fees, adjusting drug prices, or adding bonuses for case management. None of the programmes mentioned demand-side financial incentives that might affect patients’ health-seeking behaviours, such as cash transfers, vouchers, insurance, etc.

For the meso-level, most programmes kept the current fee-for-service payment system except for the ICHC programme [65], which shifted into a “pay-for-disease” for chronic diseases requiring rehabilitation but only in the outpatient setting. Three programmes were reimbursed based on a shared global budget among providers from the community (primary setting) and hospital sectors (secondary and tertiary settings). Providers were incentivised to keep patients in the primary care setting where disease prevention could avoid substantial costs, which is often cheaper and more effective than cure. Moreover, they could keep the difference between costs incurred and budgets, as they shared financial savings and losses. Two programmes (8.7%) used case-mix adjustments for reimbursements, and one insurance-based programme prepaid care providers to control the budgets better. Only two programmes from mainland China and Singapore mentioned the need for system-level re-design of the overall funding mechanisms or salary systems (8.7%).

#### Technology and medical products

About half of the programmes referred to technology and medical products (52.2%, n = 12), especially for multimorbidity patients living at home. Electronic medical records or patient portals were used at the micro-level in 40% of the programmes reviewed (n = 9). An innovative Indian mobile application (mWellcare [70,71]) allows providers to customise its functions to include 1) patients’ records, 2) supportive recommendations, and 3) monitoring functions for multimorbidity. The mobile app had IC elements of an e-health tool, assistive technology, and remote monitoring. Another platform, called “Luohu Health” [64,66], from mainland China, was available for patients to improve their health literacy. Videophone technology was also used in a Japanese programme for remote monitoring in case of falls at home. Meso-level elements were less common than micro-level, including shared information systems for appointments or imaging sharing (26.1%, n = 6) and interoperable systems (i.e., shared hospital pharmacy, referral platform) (n = 4, 17.4%). However, no macro-level elements were found.

#### Information and research

The information and research component addresses the need for collecting and collating data generated in the care process to conduct research. Some programmes (34.8%, n = 8) collected individual-level data to monitor the care process through e-health tools (i.e., mWellcare [70,71]). One programme allowed researchers to identify patient groups and predict individual risks for potential patients that could benefit from IC for resource planning. At the meso-level, one programme in Japan [69] utilised a diagnosis procedure combination (DPC) system to keep patients’ data and conduct risk stratification with more than 2500 diagnosis groups. Each patient is assigned to one of the diagnosis groups with DPC codes, reflecting their most resource-consuming disease, procedures, complications, and comorbidities. Providers were reimbursed by a flat-rate per-diem payment based on the diagnosis group, and reimbursement for the costs exceeding the specified hospitalisation period is calculated on a fee-for-service basis [77]. However, no explicit macro-level element referring to information and research was found in any of the programmes reviewed.

#### Monitoring

Monitoring was the fourth most frequently reported component to optimise the effectiveness of IC programmes (65.2%, n = 15), with the monitoring of care plans the most (47.8%, n = 11) frequently cited at the individual level. Monitoring methods included telephone follow-up, home visits, videophone consultation, short-message-system (SMS), database review, or a combination of the above approaches. Additionally, changes between face-to-face encounters in the implementation of care plans were also monitored to check potential “red flags” in home visits (8.7%, n = 2). Other programmes also followed up patients’ self-management performance (8.7%, n = 2) and clinical indicators (8.7%, n = 2).

At the meso-level, two programmes (8.7%) from mainland China and Taiwan specified quality improvement systems for quality control, managed through the administration centre and optimisation of the operational plan. At the macro-level, one top-down programme from Singapore, named Regional Health System (RHS), was structured at the system level because it was prioritised to ensure that sub-programmes were matched with the unique needs and demographics of the population.

## Discussion

This is, to our knowledge, the first scoping review to explore, map and synthesise the key components and elements mentioned in IC programmes in Asia for patients with multimorbidity. 23 IC programmes were identified. Three IC components were most frequently reported using the SELFIE framework – service delivery; leadership; and workforce – while financing was least mentioned.

Only nine (39%) IC programmes mentioned the financing component, and these mostly referred to micro-level elements such as reimbursement and financial incentives. Financial incentives were all appeared on the supply (provider) side, such as increasing funding [64,66] or adding bonuses [72] for healthcare providers. The healthcare financing systems in places where programmes were implemented in mainland China, Singapore, Taiwan, and Japan may explain this. These four countries/territories all use social health insurance systems or medical saving account as a primary source of healthcare financing. In these health financing systems, government agency acts as single purchaser and is powerful to influence the behaviour of public and private healthcare providers by introducing financial incentives. However, effective accreditation and contracting mechanisms between purchasers and providers are needed to ensure providers’ performance is accountable to people [78]. The effects of supply-side financial incentives were under-investigated due to insufficient evidence of the “financing” component from identified IC programmes.

Regarding meso-level elements such as the payment system, most IC programmes retained fee-for-service (FFS) despite higher volumes and intensity of healthcare services or procedures produced by misaligned provider behaviours [79,80,81]. FFS, being an activity-based payment system, dominates in many healthcare financing systems and encourages doctors to provide those services with higher reimbursement levels [82]. On the one hand, it neglects the need for disease prevention and long-term care that are time-consuming and have lower reimbursement costs [83], as may be required for IC. On the other hand, overtreating and overprescribing among patients with multimorbidity may be intensified under the FFS system. Although prospective payment systems such as pay-for-performance, DRGs, DPC system, and capitation have been piloted in the general population in some Asian countries (mainland China, Japan, and Taiwan) [84,85,86,87], none of them targeted multimorbidity. In some western countries, there is a shift from activity-based towards sustainable value-based payment systems such as pay-for-coordination, pay-for-performance, or global budgets [88,89,90,91,92], which might also benefit patients with multimorbidity in Asian countries as they encourage rational use of healthcare services by rewarding the parts of the healthcare delivery system that provide value (i.e., coordination of care, quality of care) [89,93].

Compared with IC programmes in EU/US countries [18,94], we found Asian countries also primarily focused on strengthening the IC delivery system and enhancing self-management [18,94]. Under the environment component, social care (IC elements of housing, community services) was less emphasised in Asian and EU/US countries [18,94], comprising 22% to 40% and 36% to 50% of IC programmes, respectively.

While the technology and medical product components were mentioned in many IC programmes in the EU/US (43% to 100%) [18,94], only 40% of IC programmes in Asia used electronic tools. The need to adopt digital technologies such as telehealth or telecare was more urgent during the recent COVID-19 pandemic [95]. As patients with multimorbidity seek services more often, using remote shared care delivery and virtual platforms can reduce the exposure of patients and treating professionals during face-to-face visits without delaying patients’ needed care [95,96].

Multidisciplinary teams in the leadership and governance component were much less prevalent in Asian IC programmes (26%) than in EU/US countries (range 50%–81%) [18,29,94]. The lack of a multidisciplinary team may be related to the current FFS payment system in Asia. When more healthcare professionals are involved in multimorbidity care, patients must pay for every single service they receive. Providers may see integration as causing a loss of income if it aims to reduce the volume of services [80]. At the meso-level, organisations need to bear the financial risk of providing services usually not reimbursed under the current FFS systems [89], creating a barrier to developing multidisciplinary teamwork.

There were very few IC programmes mentioning elements within meso- and macro-levels for all IC components, which is in line with a review by Briggs et al. studying integrated care elements for older people [29]. Mainland China, Taiwan, Singapore, Hong Kong contributed most to reporting meso- or macro-level related IC elements that require changes or reforms from the organisational or political point of view. Because these governments have the stronger political will or commitment to achieve IC reforms systematically, their involvement and leadership can facilitate the implementation of top-down IC programmes [97].

Moreover, macro-level elements were least mentioned, with only two countries, mainland China and Singapore, reporting the most. This can be attributed to the underdeveloped health systems in most Asian areas, further reflected by their varying evolution stages in healthcare financing systems. Notably, Singapore and China had considerable changes in their healthcare financing systems that could foster integrated care. Supporting by their well-established and notable “3Ms” model (Medisave, Medifund, Medishield), Singapore integrated public hospitals with community centres to enable an efficient service delivery across the patient journey. As for China, healthcare financing reform is at the core of the political agenda to shape the health system. Financial innovations such as salary reforms were piloted in some cities to stimulate the integration of care.

Regarding the effectiveness of IC programmes, nearly all of them showed satisfactory results, such as improving an individual’s quality of life, reducing readmission rates, and shortening hospital length of stay. As most of them were pilot programmes or developed in recent years, evaluation studies on their long-term effects were lacking. Moreover, more than half of the studies did not have control groups, and scarce information was related to cost-effectiveness compared to other strategies.

This paper shares several limitations common to scoping reviews. First, though the IC definition used in the screening process is aligned with the WHO definition, most articles identified did not explicitly define IC. Accordingly, we used an operational definition of IC following the Rainbow Model. Second, IC is a relatively new concept. Few IC programmes have been published in academic journals, especially for financing systems or meso- or macro-level integration strategies. Third, healthcare systems and finance vary greatly among Asian countries, so caution is needed when applying results to other systems. Lastly, due to a high proportion of out-of-pocket payments and historical legacies, the integration between public and private healthcare sectors is a unique challenge in Asia whereas not reflected in the SELFIE framework. Further study is needed to consider adding the public-private integration to modify the SELFIE framework to the Asian context.

## Conclusion

This review adopted a pragmatic scoping method to identify IC programmes for patients with multimorbidity in Asian countries. Its strength was synthesising research evidence from existing research to identify knowledge gaps and guide the future development of IC programmes [38]. First, all IC programmes emphasised the importance of distinctive service delivery; leadership; and workforce components, while the financing component was least investigated. Second, there was insufficient information about the social care provided to patients during the implementation of IC programmes. The IC component of technology and medical product, and element of the multidisciplinary team, were less reported among recognised IC programmes in Asia than EU/US countries. Last, more research on meso-level and macro-level IC elements and more cost-effectiveness evaluation analysis is needed for further development of IC programmes.

Given the potential of integrated care to address significant challenges in contemporary healthcare, we see great value in documenting experiences from many different settings in clarifying our best pathways forward.

## Additional File

The additional file for this article can be found as follows:

10.5334/ijic.6009.s1 Supplementary Material. Appendix 1–6.
