# Supplementary material for: Integrated Care for Multimorbidity Population in Asian Countries: A Scoping Review
Source: Int J Integr Care. 2022 Mar 16;22(1):22. doi: 10.5334/ijic.6009 (PMC8932356; doi:10.5334/ijic.6009)
Supplement: Supplementary Material. — Appendix 1–6. [file ijic-22-1-6009-s1.pdf]

Supplementary Contents

Appendix 1. Key concepts to identify potential studies in the scoping review 2
Appendix 2. Searching strategies 5
Appendix 3. Abbreviation list 11
Appendix 4. Characteristics of included studies 12
Appendix 5. Summary of IC programmes 28
Appendix 6. Number of IC programmes categorised by elements and components .. 36

Appendix 1. Key concepts to identify potential studies in the scoping review

Table S1. MeSH terms and keywords of concepts used in the search strategies

Concepts	MeSH terms	Keywords
**Integrated care**		Integration of care<br>Integration of health care<br>Integration of healthcare<br>Integration of patient care<br>Integration of service*<br>Care integration<br>Healthcare integration<br>health care integration<br>Service* integration<br>Integrated care<br>Integrated health care<br>Integrated healthcare<br>Integrated patient care<br>Integrated service*<br>Integrating care<br>Integrating health care<br>Integrating healthcare<br>Integrating patient care<br>Integrating service*<br>health care system integration<br>health system integration<br>provider system integration<br>coordination of care<br>care coordination<br>Healthcare coordination<br>health care coordination<br>service* coordination<br>service* co-ordination<br>coordinated care<br>coordinated health care<br>coordinated healthcare<br>coordinated patient care<br>coordinated service*<br>co-ordinated care<br>Co-ordinated service*<br>Coordinating care<br>Coordinating health care<br>Coordinating patient care<br>Coordinating service*<br>co-ordinating care<br>Co-ordinating health care<br>Co-ordinating service*<br>managed care<br>cooperative care<br>organi?ed care<br>Shar$ care<br>multidisciplinary care<br>interdisciplinary care<br>inter-disciplinary care<br>crossdisciplinary care<br>cross-disciplinary care<br>multiple interventions<br>chain* of care<br>care chain*<br>transitional care<br>care transition*

	transmural care
	horizontal integration
	vertical integration
	virtual integration
	organizational integration
	cross sectoral care
	integrated social network*
	accountable care organisations
	accountable care
	professional integration
	clinical integration
	functional integration
Continuity of patient care	Continuity of care
	Continuity of patient care
	patient care continuity
	Care continuity
	Healthcare continuity
	health care continuity
	Service* continuity
	continuum of care
	care continuum
	continuum of service*
	service* continuum
comprehensive health care	comprehensive care
	comprehensive health care
	comprehensive healthcare
	comprehensive health service
	comprehensive service*
	comprehensive patient care
Case Management	Case management
	case-management
disease management	
Delivery of Health Care, Integrated	care delivery
	delivery of health care, integrated
	delivery system*, integrated
	delivery systems, integrated
	integrated delivery system*
	integrated health care systems
	system*, integrated delivery
Patient-Centered Care	Patient centered care
patient care management	Patient centered health care
	Patient centered healthcare
	Patient centered service*
	Patient centred care
	Patient centred health care
	Patient centred healthcare
	Patient centred service*
	Patient focused care
	Patient focused health care
	Patient focused healthcare
	Patient focused service*
	population focused care
	patient care team
	population based/basis/basing care
	population based health care
	population based healthcare
	population based service*
	person centred care
	person centred health care

		person centred healthcare<br>person centred service*<br>patient-centred care<br>patient-centred health care<br>patient-centred healthcare<br>patient-centred service*<br>person-centred care<br>person-centred health care<br>person-centred healthcare<br>person-centred service*<br>comprehensive health care<br>health planning<br>care pathway*<br>Critical path*<br>medical and social healthcare<br>medical and social care<br>medical and social service*<br>health and social care<br>health and social service*
**Multimorbidity**	comorbidity<br>frail elderly	Complex chronic patients<br>frailty<br>frail elder*<br>frail older adult*<br>co-occur*<br>co occur*<br>health problem*<br>health condition*<br>chronic health problem*<br>chronic disease*<br>chronic condition*<br>chronic illness*<br>chronic disorder*<br>complex need*<br>complex condition*<br>multiple health problem*<br>multiple health condition*<br>multiple chronic condition*<br>multiple chronic disease*<br>multiple chronic disorder*<br>multiple chronic health problem*<br>multiple chronic illness*<br>multimorbid*<br>multi-morbid*<br>multi morbid*
**Asia country**		asia*<br>china<br>chinese<br>japan*<br>korea*<br>taiwan<br>hong kong<br>singapore<br>thailand<br>vietnam<br>india<br>iran<br>philippines<br>malaysia<br>Bangladesh

Appendix 2. Searching strategies

MEDLINE

Updated to 2020.06.18

Searching results: 286

1. ((integrat* or coordinat* or co-ordinat* or managed or cooperative or organi?ed or shar* or comprehensive or multidisciplinary or interdisciplinary or inter-disciplinary or crossdisciplinary or cross-disciplinary or transition* or transmural or "cross sectoral" or continuity or continuum or ((patient or patient- or person or person- or population or population- or people or people-) adj2 (cent?red or focus* or bas*))) adj4 (service* or care or "health care" or health-care or "patient care" or healthcare)).ti,ab.

2. ((integrat* or coordinat* or co-ordinat* or managed or cooperative or organi?ed or shar* or comprehensive or multidisciplinary or interdisciplinary or inter-disciplinary or crossdisciplinary or cross-disciplinary or transition* or transmural or "cross sectoral" or continuity or continuum or ((patient or patient- or person or person- or population or population- or people or people-) adj2 (cent?red or focus* or bas*))) adj4 ((service* or care or "health care" or health-care or "patient care" or healthcare) adj3 (system* or "provider system"))).ti,ab.

3. multiple intervention*.ti,ab.

4. (chain* adj3 (care or "health care" or healthcare or health-care)).ti,ab.

5. ((horizontal or vertical or virtual or organi?ational or professional or clinical or functional) adj3 integration).ti,ab.

6. "integrated social networks".ti,ab.

7. (accountable adj2 (care or "health care" or healthcare or health-care)).ti,ab.

8. ("case management" or case-management).ti,ab.

9. ("care delivery" or ("delivery of" adj2 "health care" adj2 integrate*) or ("delivery system" adj2 integrat*) or ("delivery systems" adj2 integrat*) or (integrat* adj2 "delivery system") or (integrat* adj2 "delivery systems") or (integrat* adj2 "health care system") or (integrat* adj2 "health care systems") or (integrat* adj2 "healthcare system") or (integrat* adj2 "healthcare systems") or (system* adj2 integrat* adj2 delivery) or ("delivery of" adj2 "healthcare system") or "integrated healthcare delivery" or "integrated health care delivery" or "integrated care delivery").ti,ab.

10. "patient care team".ti,ab.

11. "health planning".ti,ab.

12. (care pathway* or critical path*).ti,ab.

13. "Delivery of Health Care, Integrated"/

14. "Continuity of Patient Care"/

15. Comprehensive Health Care/

16. Case Management/

17. Disease Management/

18. Patient-Centered Care/

19. Patient Care Management/

20. ("medical and social healthcare" or "medical and social care" or ("medical and social" adj2 service*) or "health and social care" or ("health and social" adj2 service*)).ti,ab.

21. (("multiple chronic" or "complex chronic") adj2 (condition* or disease* or disorder* or "health problem" or "health problems" or illness*)).ti,ab.

22. ("multiple health" adj2 (problem* or condition*)).ti,ab.

23. Frail Elderly/

24. Comorbidity/

25. (frailty or (frail adj2 elder*) or ((frail adj2 older) and adult) or ((frail adj2 older) and adults)).ti,ab.

26. ((co-occur* or co occur* or "co occurring" or "co occurrent" or "co occurrence" or multiple) adj3 (("chronic health" adj2 problem*) or disorder* or (health adj2 problem*) or (chronic adj2 (disease* or condition* or illness*)))).ti,ab.

27. (complex adj2 (need* or condition*)).ti,ab.

28. (multimorbid* or multi-morbid* or multi morbid*).ti,ab.

29. 1 or 2 or 3 or 4 or 5 or 6 or 7 or 8 or 9 or 10 or 11 or 12 or 13 or 14 or 15 or 16 or 17 or 18 or 19 or 20

30. 21 or 22 or 23 or 24 or 25 or 26 or 27 or 28

31. exp Asia/ or asia.mp.

32. (china or chinese or japan* or korea* or taiwan or hong kong or singapore or thailand or vietnam or india* or iran or philippines or malaysia or Bangladesh).mp.

33. exp china/ or exp Japan/ or exp korea/ or exp taiwan/ or exp hong kong/ or exp singapore/ or exp thailand/ or exp vietnam/ or exp india/ or exp iran/ or exp philippines/ or exp malaysia/ or exp Bangladesh/

34. 31 or 32 or 33

35. 29 and 30 and 34

36. (animal studies or animals, laboratory or experimental animal or animal experiment or animal model or rodentia or rodents or rodent).sh.

37. (editorial or historical article or anecdote or comment or commentary or note or case report* or case study or newspaper article or news or letter*).pt.

38. 36 or 37

39. 35 not 38

40. limit 39 to human

EMBASE

Updated to 2020.06.18

Searching resulst: 377

1. ((integrat* or coordinat* or co-ordinat* or managed or cooperative or organi?ed or shar* or comprehensive or multidisciplinary or interdisciplinary or inter-disciplinary or crossdisciplinary or cross-disciplinary or transition* or transmural or "cross sectoral" or continuity or continuum or ((patient or patient- or person or person- or population or population- or people or people-) adj2 (cent?red or focus* or bas*))) adj4 (service* or care or "health care" or health-care or "patient care" or healthcare)).ti,ab.

2. ((integrat* or coordinat* or co-ordinat* or managed or cooperative or organi?ed or shar* or comprehensive or multidisciplinary or interdisciplinary or inter-disciplinary or crossdisciplinary or

cross-disciplinary or transition* or transmural or "cross sectoral" or continuity or continuum or ((patient or patient- or person or person- or population or population- or people or people-) adj2 (cent?red or focus* or bas*))) adj4 ((service* or care or "health care" or health-care or "patient care" or healthcare) adj3 (system* or "provider system"))).ti,ab.

3. multiple intervention*.ti,ab.

4. (chain* adj3 (care or "health care" or healthcare or health-care)).ti,ab.

5. ((horizontal or vertical or virtual or organi?ational or professional or clinical or functional) adj3 integration).ti,ab.

6. "integrated social networks".ti,ab.

7. (accountable adj2 (care or "health care" or healthcare or health-care)).ti,ab.

8. ("case management" or case-management).ti,ab.

9. ("care delivery" or ("delivery of" adj2 "health care" adj2 integrate*) or ("delivery system" adj2 integrat*) or ("delivery systems" adj2 integrat*) or (integrat* adj2 "delivery system") or (integrat* adj2 "delivery systems") or (integrat* adj2 "health care system") or (integrat* adj2 "health care systems") or (integrat* adj2 "healthcare system") or (integrat* adj2 "healthcare systems") or (system* adj2 integrat* adj2 delivery) or ("delivery of" adj2 "healthcare system") or "integrated healthcare delivery" or "integrated health care delivery" or "integrated care delivery").ti,ab.

10. "patient care team".ti,ab.

11. "health planning".ti,ab.

12. (care pathway* or critical path*).ti,ab.

13. integrated health care system/

14. long term care/ or case management/ or patient care planning/

15. disease management/

16. ("medical and social healthcare" or "medical and social care" or ("medical and social" adj2 service*) or "health and social care" or ("health and social" adj2 service*)).ti,ab.

17. (("multiple chronic" or "complex chronic") adj2 (condition* or disease* or disorder* or "health problem" or "health problems" or illness*)).ti,ab.

18. ("multiple health" adj2 (problem* or condition*)).ti,ab.

19. (frailty or (frail adj2 elder*) or ((frail adj2 older) and adult) or ((frail adj2 older) and adults)).ti,ab.

20. ((co-occur* or co occur* or "co occurring" or "co occurrent" or "co occurrence" or multiple) adj3 (("chronic health" adj2 problem*) or disorder* or (health adj2 problem*) or (chronic adj2 (disease* or condition* or illness*)))).ti,ab.

21. (complex adj2 (need* or condition*)).ti,ab.

22. (multimorbid* or multi-morbid* or multi morbid*).ti,ab.

23. frail elderly/

24. multiple chronic conditions/

25. 1 or 2 or 3 or 4 or 5 or 6 or 7 or 8 or 9 or 10 or 11 or 12 or 13 or 14 or 15

26. 16 or 17 or 18 or 19 or 20 or 21 or 22 or 23 or 24

27. exp Asia/ or asia.mp.

28. (china or chinese or japan* or korea* or taiwan or hong kong or singapore or thailand or vietnam or india* or iran or philippines or malaysia or Bangladesh).mp.

29. exp china/ or exp Japan/ or exp korea/ or exp taiwan/ or exp hong kong/ or exp singapore/ or exp thailand/ or exp vietnam/ or exp india/ or exp iran/ or exp philippines/ or exp malaysia/ or exp Bangladesh/

30. 27 or 28 or 29

31. 25 and 26 and 30

32. (animal studies or animals, laboratory or experimental animal or animal experiment or animal model or rodentia or rodents or rodent).sh.

33. (editorial or historical article or anecdote or comment or commentary or note or case report* or case study or newspaper article or news or letter*).pt.

34. 32 or 33

35. 31 not 34

36. limit 35 to human

Scopus

Updated to 2020.06.18

Searching resulst: 45

(TITLE-ABS-KEY(((integrat* or coordinat* or co-ordinat* or managed or cooperative or organi?ed or shar* or comprehensive or multidisciplinary or interdisciplinary or inter-disciplinary or crossdisciplinary or cross-disciplinary or transition* or transmural or "cross sectoral" or continuity or continuum or ((patient or patient- or person or person- or population or population- or people or people-) W/1 (cent?red or focus* or bas*))) W/3 (service* or care or "health care" or health-care or "patient care" or healthcare)) or ((integrat* or coordinat* or co-ordinat* or managed or cooperative or organi?ed or shar* or comprehensive or multidisciplinary or interdisciplinary or inter-disciplinary or crossdisciplinary or cross-disciplinary or transition* or transmural or "cross sectoral" or continuity or continuum or ((patient or patient- or person or person- or population or population- or people or people-) W/1 (cent?red or focus* or bas*))) W/3 ((service* or care or "health care" or health-care or "patient care" or healthcare) W/2 (system* or "provider system"))) or "multiple interventions" or (chain* W/2 (care or "health care" or healthcare or health-care)) or ((horizontal or vertical or virtual or organi?ational or professional or clinical or functional) W/2 integration) or "integrated social networks" or (accountable W/1 (care or "health care" or healthcare or health-care)) or "case management" or case-management or "care delivery" or (“delivery of” W/1 “health care” W/1 integrat*) or ("delivery system” W/1 integrat*) or ("delivery systems” W/1 integrat*) or (integrat* W/1 “delivery system") or (integrat* W/1 “delivery systems") or (integrat* W/1 “health care system") or (integrat* W/1 “health care systems") or (system* W/1 integrat* W/1 delivery) or ("delivery of” W/1 “healthcare system") or ("delivery of” W/1 “healthcare systems") or (integrat* W/1 “healthcare system") or "integrated healthcare delivery" or "integrated health care delivery" or "integrated care delivery" or "patient care team" or "health planning" or care pathway* or critical path* or "continuity of care" or "continuity of patient care" or "patient care continuity" or "care continuity" or "healthcare continuity" or "health care continuity" or "service continuity" or "services continuity" or ("continuum of” W/1 care) or "care continuum" or ("continuum of” W/1 service*) or "service continuum" or "services continuum" or (comprehensive W/1 care) or (comprehensive W/1 “health care") or (comprehensive W/1 healthcare) or (comprehensive W/1 service*) or "case management" or case-management or "disease management")) AND (TITLE-ABS-KEY(multimorbid* OR (multi W/1 morbid*) OR (“multiple chronic” or "complex

chronic" W/1 (condition* OR disease* OR disorder* OR “health problem” OR “health problems” OR illness*)) OR (“multiple health” W/1 (problem* OR condition*)) OR (complex W/1 (condition* OR need*)) OR ((o-occur* OR “co occurring” OR “co occurrent” OR “co occurrence”) W/2 “chronic health” W/1 problem*) OR ((o-occur* OR “co occurring” OR “co occurrent” OR “co occurrence”) W/2 disorder*) OR ((o-occur* OR “co occurring” OR “co occurrent” OR “co occurrence”) W/2 health W/1 problem*) OR ((o-occur* OR “co occurring” OR “co occurrent” OR “co occurrence”) W/2 chronic W/1 (disease* OR condition* OR illness)) OR frailty OR (frail W/1 elder*) OR ((frail W/1 older) AND adult) OR ((frail W/1 older) AND adults))) AND NOT (DOCTYPE(ed or er or le or no or pr or re or sh))

AND (CU=(china or chinese or japan* or korea* or taiwan or hong kong or singapore or thailand or vietnam or india* or iran or philippines or malaysia or Israel or asia*) or AD=(china or chinese or japan* or korea* or taiwan or hong kong or singapore or thailand or vietnam or india* or iran or philippines or malaysia or Israel or asia*) or TS=(china or chinese or japan* or korea* or taiwan or hong kong or singapore or thailand or vietnam or india* or iran or philippines or malaysia or Israel or asia*))

Web of Science

Updated to 2020.06.18

Searching resulst: 390

(TS=(((integrat* or coordinat* or co-ordinat* or managed or cooperative or organi?ed or shar* or comprehensive or multidisciplinary or interdisciplinary or inter-disciplinary or crossdisciplinary or cross-disciplinary or transition* or transmural or "cross sectoral" or continuity or continuum or ((patient or patient- or person or person- or population or population- or people or people-) NEAR/1 (cent?red or focus* or bas*))) NEAR/3 (service* or care or "health care" or health-care or "patient care" or healthcare)) or ((integrat* or coordinat* or co-ordinat* or managed or cooperative or ordain?ed or shar* or comprehensive or multidisciplinary or interdisciplinary or inter-disciplinary or crossdisciplinary or cross-disciplinary or transition* or transmural or "cross sectoral" or continuity or continuum or ((patient or patient- or person or person- or population or population- or people or people-) NEAR/1 (cent?red or focus* or bas*))) NEAR/3 ((service* or care or "health care" or health-care or "patient care" or healthcare) NEAR/2 (system* or "provider system"))) or "multiple interventions" or (chain* NEAR/2 (care or "health care" or healthcare or health-care)) or ((horizontal or vertical or virtual or organisational or organizational or professional or clinical or functional) NEAR/2 integration) or "integrated social networks" or (accountable NEAR/1 (care or "health care" or healthcare or health-care)) or "case management" or case-management or "care delivery" or (("delivery of" NEAR/1 "health care") NEAR/1 integrat*) or ("delivery system" NEAR/1 integrat*) or ("delivery systems" NEAR/1 integrat*) or (integrat* NEAR/1 "delivery system") or (integrat* NEAR/1 "delivery systems") or (integrat* NEAR/1 "health care system") or (integrat* NEAR/1 "health care systems") or (system* NEAR/1 integrat* NEAR/1 delivery) or ("delivery of" NEAR/1 "healthcare system") or (integrat* NEAR/1 "healthcare system") or "patient care team" or "health planning" or care pathway* or critical path* or "continuity of care" or "continuity of patient care" or "patient care continuity" or "care continuity" or "healthcare continuity" or "health care continuity" or "service continuity" or "services continuity" or ("continuum of" NEAR/1 care) or "care continuum" or ("continuum of" NEAR/1 service*) or "service continuum" or "services continuum" or (comprehensive NEAR/1 care) or

(comprehensive NEAR/1 "health care") or (comprehensive NEAR/1 healthcare) or (comprehensive NEAR/1 service*) or "case management" or case-management or "disease management") **AND** (TS=(multimorbid* OR (multi NEAR/1 morbid*) OR (“multiple chronic” or "complex chronic" NEAR/1 (condition* OR disease* OR disorder* OR “health problem” OR “health problems” OR illness*)) OR (“multiple health” NEAR/1 (problem* OR condition*)) OR (complex NEAR/1 (condition* OR need*)) OR ((o-occur* OR “co occurring” OR “co occurrent” OR “co occurrence”) NEAR/2 “chronic health” NEAR/1 problem*) OR ((o-occur* OR “co occurring” OR “co occurrent” OR “co occurrence”) NEAR/2 disorder*) OR ((o-occur* OR “co occurring” OR “co occurrent” OR “co occurrence”) NEAR/2 health NEAR/1 problem*) OR ((o-occur* OR “co occurring” OR “co occurrent” OR “co occurrence”) NEAR/2 chronic NEAR/1 (disease* OR condition* OR illness)) OR frailty OR (frail NEAR/1 elder*) OR ((frail NEAR/1 older) AND adult) OR ((frail NEAR/1 older) AND adults))) **NOT** (DT= (Editorial Material OR Letter or News Item OR Note))

AND (CU=(china or chinese or japan* or korea* or taiwan or hong kong or singapore or thailand or vietnam or india* or iran or philippines or malaysia or Israel or asia*) or AD=(china or chinese or japan* or korea* or taiwan or hong kong or singapore or thailand or vietnam or india* or iran or philippines or malaysia or Israel or asia*) or TS=(china or chinese or japan* or korea* or taiwan or hong kong or singapore or thailand or vietnam or india* or iran or philippines or malaysia or Israel or asia*))

Appendix 3. Abbreviation list

Table S2. Abbreviation list for identified IC programmes

Abbreviation	Name of IC programme
ACTION	The Aged Care Transition Programme
AMU	Acute Medical Unit programme
CM	Case Management programme
EPFU	Emergency Physician–led Frailty-care Unit programme
FDP	Family Doctor Plan programme
HDS	Hospital Discharge Services programme
HSTCM	Health-Social Transitional Care Management Programme
ICCS	Integrated Community Care System programme
ICHC	Integrated County Healthcare Consortium programme
JHC	Joint Health Centre for chronic care programme
Luohu Group	Integrated Service Delivery Network-Luohu Hospital Group programme
mWellcare	mWellcare Programme - mHealth-based electronic decision support programme
NCM	Nurse-led Case Management Programme
(NUHS-RHS) NICE	National University Health System-Regional Health System Integrated Interventions and Care Extension programme
(NUHS-RHS) TC	National University Health System-Regional Health System Transitional Care Programme
RHS	Regional Health System programme
RSC	Right-Site Care programme
SGH-TC	Singapore General Hospital Transitional Care Programme
SGH-THC	Singapore General Hospital transitional home care programme
TCM	Targeted Case Management programme
THC-IPU	Transitional Home Care-Integrated Practice Units Programme
TPPCT	Training Programmes for Primary Care Teams programme
VP	Home Healthcare Services Provided with Videophones programme

Appendix 4. Characteristics of included studies

Table S3. Summary of Studies Included in Scoping Review

First author (year)	Paper title	Countr y/region	Aim(s) and study design	Participants and care setting	Intervention (and control) description	Results
**Nakamura, K., et al.(1999)**	*The effectiveness of videophones in home healthcare for the elderly.*	JP	To evaluate the effectiveness of telecare, the use of videophones in healthcare for the elderly in communities, and propose an effective application of telecare in home healthcare.<br><br>**Mixed methods:** trail and group interview	32 eligible cases enrolled and 13 professionals (group interview)<br><br>home setting	**Intervention: home healthcare services provided with videophones (n=16)**<br>Services including: (a) medical consultation, (b) exercise instructions, (c) comprehensive assessments and instructions regarding to patients and caregiving, (d) advice and instructions for healthcare workers, (e) advice on the effective use of health and welfare resources, (f) emotional support.<br><br>**Control: regular home healthcare services (n=16)**	Significant improvement in ADL, communication, and social cognition independence of the intervention group over the 3-month trial period than those of the control group.<br><br>The responses of home healthcare professionals showed definite potential benefits in terms of improving communication skills, offered better assistance to expand clients' everyday activities and social activities, providing fact-based assessments regarding clients' daily living, offering better emotional support for the clients and their families, helping communication between clients to facilitate peer counselling, confirming the safety of clients living alone, and improving communications among home healthcare professionals.
**Leung, A.C.T., et al.(2004)**	*Reducing utilisation of hospital services by case management: a randomised controlled trial.*	HK	To evaluate the effectiveness of case management in terms of utilisation of hospital services.<br><br>**Quantitative method:** randomized controlled trial	92 eligible patients and their caregivers recruited from a hospital<br><br>ambulatory setting<br>community setting<br>home setting (mainly)	**Intervention: Targeted Case Management (n=45)**<br>Services including: (a) regular monitoring of subjects' health status, (b) daily phone assistance , (c) home visits, (d) prescribing of community-based supportive services (i.e. community nursing services), (e) access to the case geriatrician by the case manager for medical support (telephone consultation, assessment of subjects in the outpatient department, and admission of subjects to the hospital).<br><br>**Control: usual care (n=47)**<br>Usual services of regular medical follow-up	(a) lower mean total number of hospital bed-days;<br>(b) lower mean total episodes of hospital admissions;<br>(c) lower mean total number of attendances at the outpatient department

**Leung, A.C.T., et al.(2004)**	*Cost-benefit analysis of a case management project for the community-dwelling frail elderly in Hong Kong.*	HK	To evaluate the cost-benefit of a case management project for elderly in Hong Kong.<br><br>**Quantitative method:** randomized controlled trial	260 eligible patients<br><br>home setting<br>community setting	**Intervention: Case Management (n=130)**<br>Services including:(a) home visit and telephone follow-up, (b) comprehensive geriatric assessment, (c) development of care plan, (d) coordinate health and social services, (e) monthly monitoring via IT system, (f) on-site and/or phone health and psychosocial counselling, (g) health educational programmes for patients and caregivers.<br><br>**Control: usual medical care (n=130)**<br>Conventional health and social services	All participants had improved significantly in their levels of mood symptoms ($p < .001$) and informal support ($p < .001$).<br><br>Greater improvement in the level of continence, reduced the total number of unplanned hospital admissions, acute hospital bed days than those older persons in the control group.<br><br>The total savings over time in overall health care costs for the intervention group was still 93% more than that in the control group (U.S.$179,090 vs. U.S.$12,526).
**Yau, D.C., et al.(2005)**	*Global case management: Hong Kong. Care for the hospital-discharged frail elders by nurse case managers: a process evaluation of a longitudinal case management service project.*	HK	To (i) have an understanding of the case management activities performed by nurse case managers (NCMs) and (ii) identify essential factors for a successful case management service.<br><br>**Mixed methods:** a longitudinal study	45 patients' records and 4 NCMs (interviews)<br><br>ambulatory setting<br>community setting<br>home setting (mainly)	**Intervention: Targeted Case Management (n=45)**<br>Services including: (a) regular monitoring of subjects' health status, (b) daily phone assistance , (c) home visits, (d) prescribing of community-based supportive services (i.e. community nursing services), (e) access to the case geriatrician by the case manager for medical support (telephone consultation, assessment of subjects in the outpatient department, and admission of subjects to the hospital).<br><br>**Control: usual care (n=47)**<br>Usual services of regular medical follow-up	The common reasons for client-initiated telephone calls to NCMs and NCMs' interventions to these calls. Qualitative data yielded 9 major themes on which a sequential and dynamic process model of case management was conceptualized. Another 7 thematic descriptions on essential factors for the successful implementation of case management were configured in a dual-dimensional framework of staff and structural factors.
**Wong, F., et al.(2011)**	*Effects of a health-social partnership transitional program on hospital readmission: A randomized controlled trial.*	HK	To explore the outcomes of a health-social partnership program on post-discharge medical patients.<br><br>**Quantitative method:** randomized controlled trial	555 eligible patients recruited through an acute regional hospital<br><br>home setting	**Intervention: Health-Social Transitional Care Management Programme (n=272)**<br>Service including: (a) pre-discharge phase: pre-discharge assessment, (b) post-discharge phase: first week - the NCM and TVs conducted a home visit together, second week - the NCM made a telephone follow-up call, third week - the TVs conducted a home visit in pairs, fourth week - the NCM made the final telephone follow-up call.<br><br>**Control: usual discharge care (n=283)**	The programme significantly reduced readmission at 4-weeks (study 4.0%, control 10.2%, $p=0.005$). The intention-to-treat result also showed a lower readmission rate with the study group but the result was not significant (study 11.5%, control 14.7%, $p= 0.258$). There was however significant improvement in quality of life, self-efficacy and satisfaction in the study group in both per-protocol and intention-to-treat analyses.

**Wong, F., et al.(2012)**	*Cost-effectiveness of a health-social partnership transitional program for post-discharge medical patients.*	HK	To exam the cost-effectiveness of a health-social partnership transitional care program for patients discharged from hospitals.<br><br>**Quantitative method:** randomized controlled trial	555 eligible patients recruited through an acute regional hospital<br><br>home setting	**Intervention: Health-Social Transitional Care Management Programme (n=272)**<br>Service including: (a) home visit, (b) assessment in the domains of environment, physical, psychosocial and health-related behaviours and provide relevant intervention, (c) social support, (d) follow-up telephone calls, (e) further social assessment and interventions (daily living assistance, housing assistance, and counselling), (f) regular case reviews.<br><br>**Control: usual discharge care (n=283)**	The readmission rates within 28 (control 10.2%, study 4.0%) and 84 days (control 19.4%, study 8.1%) were significantly higher in the control group.<br><br>Utility values showed no difference between the control and study groups at baseline ($p = 0.308$). Utility values for the study group were significantly higher than in the control group at 28 ($p < 0.001$) and 84 days ($p = 0.002$). The study group also had a significantly higher QALYs gain ($p < 0.001$) over time at 28 and 84 days when compared with the control group.<br><br>The cost of readmission per subject within 28 and 84 days was lower in the study group than in the control group, and the differences were -HK$1505 (95% CI: -$2670, -$555) and -HK$3000 (−$5104, -$1211) for the two time periods respectively.<br><br>The intervention had an 89% chance of being cost-effective at the threshold of £20000/QALY.
**Chow, S.K.Y.,et al.(2014)**	*A randomized controlled trial of a nurse-led case management programme for hospital-discharged older adults with co-morbidities.*	HK	To examine the effects of a nurse-led case management programme for hospital- discharged, older adults with co-morbidities in Hong Kong.<br><br>**Quantitative method:** randomized controlled trial	281 eligible patients recruited from a hospital<br><br>home setting	**Intervention: arm 1-home visit (n=98)**<br>Services including: (a) comprehensive pre-discharge needs assessment by NCM, (b) development of goal and care plan with patients, (c) analysis of performance barriers with patients, (d) monitoring of system (regular meeting, process evaluation), (e) home visit for post-discharge assessment, (f) training of nursing students, (g) closing call to motivate and support the patients in maintaining self-management behaviours.<br><br>**Intervention: arm 2-call (n=96)**<br>Same services as arm 1 excluding home visit service.<br><br>**Control: arm 3-usual care(n=98**)<br>usual care and social calls (topic about weather, television programmes, etc.)	(a) lower readmission rates;<br>(b) better self-rated health and self-efficacy:<br>(c) significant difference between the groups in the physical composite score;<br>(d) no significant difference in mental component score in SF-36 scale

**Wee, S.L., et al.(2014)**	*Effectiveness of a National Transitional Care Program in Reducing Acute Care Use.*	SG	To evaluate the effectiveness of a national transitional care program for elderly adults with complex care needs and limited social support.<br><br>**Quantitative method:** retrospective cohort study	8,264 cases enrolled<br><br>inpatient setting home setting	**Intervention: The Aged Care Transition Programme (n=4,132)**<br>Services including: (a) comprehensive assessment, (b) medical services, (c) social services, (d) self-management support, (e) care planning, (f) follow-up through calls and home visits, (g) coordinate referral.<br><br>**Control: non-programme user (n=4,132)**	Recipients of the programme had fewer unplanned rehospitalizations and emergency departments (EDs) visits after discharge. Propensity score–adjusted odds ratios of participants versus control for number of unplanned rehospitalization and ED visits were 0.5 (95% confidence interval (CI) = 0.5–0.6) and 0.81 (95% CI = 0.72–0.90) 30 days after discharge and 0.6 (95% CI = 0.6–0.7) and 0.90 (95% CI = 0.82–0.99) 180 days after discharge. Quality of life and self-rated health were better 4 to 6 weeks after discharge than 1 week after discharge.
**Lee, K.H., et al.(2015)**	*Transitional care for the highest risk patients: findings of a randomised control study.*	SG	To (i) find out if the programme could reduce the rate of unscheduled readmissions and (ii) determine if the proposed transitional care programme improved emergency department attendance rates and patient satisfaction.<br><br>**Quantitative method:** randomized controlled trial	840 eligible patients<br><br>ambulatory setting home setting	**Intervention: Singapore General Hospital Transitional Care Programme (n=419)**<br>Services including:(a) post-discharge surveillance of the patient to ensure adherence to care plans, (b) coordination of follow-up visits with specialist care providers, (c) patient education and caregiver coaching, (d) activation of community and social services.<br><br>**Control: usual medical care (n=421)**<br>Services including: 2h education on-site course as the intervention group	Proportion of patients with readmission within 60 and 90 days after intervention were not statistically significant between the intervention and control groups.<br><br>The rate of emergency department visits within 30, 60 and 90 days did not differ significantly between the intervention and control groups.<br><br>The intervention group reported a significantly higher level of satisfaction in all aspects of care surveyed.

**Nakanishi, M., et al.(2015)**	*"Ageing in Place" Policy in Japan: Association Between the Development of an Integrated Community Care System and the Number of Nursing Home Placements Under the Public Long-Term Care Insurance Program Among Municipal Governments.*	JP	To examine the association between progress in developing an Integrated Community Care system and the number of nursing home placements under the Long Term Care Insurance (LTCI) program among municipal governments in Japan.<br><br>**Quantitative method:** retrospective longitudinal study	1,738 municipalities targeted with 612 (35.2%) responded<br><br>community setting	**Intervention: Integrated Community Care System (n=612)**<br>multidisciplinary service networks: (a) prevention of long-term care, (b) support for case managers, (c) case conference, (d) discharge planning, (e) home healthcare, (f) end of life care at home, (g) housing provision, (h) improved home environment, (i) monitoring elderly, (j) prevention of social isolation, (k) suicide prevention, (l) disaster risk management, (m) advocacy for elderly, (n) elder abuse prevention and intervention.<br><br>**Control: none**	A significantly smaller annual numbers of nursing home placements per 1000 elderly persons among municipalities that had larger number of agencies joining in multiple service networks of the Integrated Community Care system.<br><br>A significantly greater number of nursing home placements was also observed among municipalities that had a larger elderly population rate, lower annual number of elderly individuals in intermediate care per 1000 elderly persons, and smaller number of elderly individuals in congregate housings per 1000 elderly persons.

**Prabhakaran, D., et al.(2019)**	*Effectiveness of an mHealth-Based Electronic Decision Support System for Integrated Management of Chronic Conditions in Primary Care: The mWellcare Cluster-Randomized Controlled Trial.*	ID	To evaluate the effectiveness of a nurse-facilitated, mHealth-based EDS system for the integrated management of 5 chronic conditions in primary care settings of India through the mWellcare trial.<br><br>**Quantitative method:** cluster-randomized controlled trial	3,698 eligible patients across 40 clusters enrolled with 3324 completed the trial<br><br>community setting	**Intervention: mWellcare Programme - mHealth-based electronic decision support (EDS) system (n=20 clusters, 1,856 patients)**<br>Services including: **for EDS system:** (a) generate EDS recommendations for the management of diseases, (b) store electronic health records, (c) long-term monitoring and follow-up, (d) reminder message service for scheduled medication adherence and follow-up visits; **for training:** (e) centralized training on the clinical management guidelines to all physicians , (f) onsite training for NCD nurses in the management of hypertension, diabetes mellitus, depression, and tobacco and alcohol use, (g) training, onsite supervisor and support for nurses for orientation of the system.<br><br>**Control: enhanced usual care (EUC) (n=20 clusters, 1,842 patients)**<br>Services including: **training:** (a) training to physicians on the clinical management guidelines for hypertension and diabetes mellitus, (b) training to NCD nurses in the management of hypertension and diabetes mellitus, (c) EUC NCD nurses with a tablet computer (without the mWellcare system) for collecting data	No evidence of difference between the 2 arms for systolic blood pressure ("=−0.98; 95% CI, −4.64 to 2.67) and glycated haemoglobin ("=0.11; 95% CI, −0.24 to 0.45) even after adjustment of several key variables.<br><br>Similarly, there were no differences in the changes between the 2 groups for tobacco and alcohol use or other secondary outcomes.

**Low, L.L., et al.(2015)**	*Effectiveness of a transitional home care program in reducing acute hospital utilization: a quasi-experimental study.*	SG	To evaluate the effectiveness of a transitional home care program in reducing hospital admissions and emergency department attendances of medically complex patient.<br><br>**Quantitative method:** quasi-experimental study (pre and post analysis)	262 eligible patients were enrolled into the program and 259 were analysed<br><br>home setting	**Intervention: Singapore General Hospital Transitional Home Care Programme (n=259)**<br>Service including: (a) comprehensive medical and nursing assessment within the first week of discharge (1. Optimizing medical conditions in the home setting, 2. Educating patients and caregivers on self-management of chronic diseases by using action plans, 3. Reducing polypharmacy and medication conflicts through medication reconciliation and facilitating adherence to treatment, 4. Ensuring appropriate follow up by coordination of care with hospital specialists, 5. Activating appropriate community services to support the patient's care in the home setting), (b) draw up individualized patient-centred care plan, (c) telephone call reviews, (d) early physician reviews.<br><br>**Control: none**	Patients had a 51.6% and 52.8% reduction ($p < 0.001$) in hospital admissions in the three-month and six-month post enrolment, respectively. Similarly, a 47.1% and 48.2% reduction ($p < 0.001$) was observed for emergency department attendances in the three and six months post enrolment, respectively. The average difference in per patient hospital bed days in the pre- and post-enrolment periods were 12.05 days ($p = 0.004$) and 20.03 days ($p=0.869$) at the 3-month and 6-month periods, respectively.
**Low, L.L., et al.(2017)**	*Transitional Home Care Program Utilizing the Integrated Practice Unit Concept (THC-IPU): Effectiveness in Improving Acute Hospital Utilization.*	SG	To (i) evaluate the effectiveness of THC-IPU in reducing 30-day readmission and improve acute hospital utilization within 30 days and 90 days of enrolment into the program, and (ii) identify patient subgroups that will benefit maximally from the THC-IPU program.<br><br>**Quantitative method:** retrospective cohort study	1,166 eligible patients recruited from the hospital<br><br>home setting	**Intervention: Transitional Home Care-Integrated Practice Units Programme (n=541)**<br>Service including: (a) comprehensive medical and nursing assessment within two weeks of discharge, (b) medication reconciliation, (c) patient and caregiver education using standardized action plans and videos, (d) setting up a personal health record, (e) multi-disciplinary team meetings, (f) dedicated nurse to do telephone follow up to maintain continuity of care, (g) allied health services, (h) subsequent routine or subacute home visits, (i) readmissions review.<br><br>**Control: non-programme users (n=625)**	The differences in percentage between control and intervention for admission (19.2% vs 13.1%) and ED attendances (19.8% vs 15.2%) are statistically significant ($p = 0.005$ and 0.037 respectively) whereas the differences for SOC attendances (45.8% vs 43.3%) is not statistically significant ($p = 0.390$). The results are consistent after using the model building approach to adjust for confounders.<br><br>For 30 days admission, patients with higher Charlson Score have lower odds ratios than patients with Charlson Score of 0 to 2 (OR 0.93; 95% CI 0.55 – 1.54). Patients in both subgroups of Charlson Score 3 to 5 and Charlson Score more than 5 have significant odds ratio of 0.46 (95% CI 0.26 – 0.81) and 0.43 (95% CI 0.20 – 0.93) respectively.<br><br>There were three significant interaction terms for the outcome of ED attendances, namely for different levels of number of admissions 30 days before index hospitalization ($p = 0.024$), Charlson Score ($p = 0.047$), and gender ($p = 0.007$)

**Qian, Y., et al.(2017)**	*Integrated care reform in urban China: a qualitative study on design, supporting environment and implementation.*	CN	To identify and solve the constraining factors in the future implementation and scale up the integrated care re- form in China and in other countries with similar context.<br><br>**Qualitative method:** in-depth interviews and focus group discussions	50 key informants (policy makers, administrative staff and medical staff) were purposely recruited<br><br>ambulatory setting<br>inpatient setting<br>community setting	**Intervention: Joint Health Center pilot programme**<br>Service including: (a) screening services, (b) consulting services, (c) follow-up services, (d) referral through an E-medical appointment system.<br><br>**Control: none**	The pilot in Hangzhou was established as a Community Health Centre (CHC)-led delivery system based on cooperation agreement between CHCs and hospitals to deliver primary and specialty care together for patients with chronic diseases. An innovative learning-from-practice mentorship system between specialists and general practitioners was also introduced to solve the poor capacity of general practitioners. The design of the pilot, its governance and organizational structure and human resources were enabling factors, which facilitated the integrated care reform. However, the main constraining factors were a lack of an integrated payment mechanism from health insurance and a lack of tailored information system to ensure its sustainability.
**Goh, W.P., et al.(2018)**	*Acute medical unit: experience from a tertiary healthcare institution in Singapore.*	SG	To describe the process, outcomes and learning points from AMU development initiative.<br><br>**Qualitative method:** descriptive study	N/A<br><br>inpatient setting	**Intervention: Acute Medical Unit**<br>Services including: (a) early internist-led assessment and management, (b) holistic management, (c) active re-triaging of patient, (d) inpatient treatment triggered, (e) allied health services, (f) clinical management support, (g) clinical services support.<br><br>**Control: none**	The internists provide holistic, patient-centric care with better overall ownership of the patients, improved efficiency and less fragmentation of care.<br>The model of care also addresses conditions in which management plans can be expedited through having rapid access to investigations.<br>It allows the specialty inpatient clinical teams to concentrate on the care of patients who truly need advanced tertiary care.<br>It helps the hospital to rationalise resource allocation.<br>It led to better containment and significantly reduced scatter of medical patients, especially to surgical wards.<br>It serves as a good learning ground for residents and medical students with its remarkable case mix and exposure to acute medicine.

**Jindal, D., et al.(2018)**	*Development of mWellcare: an mHealth intervention for integrated management of hypertension and diabetes in low-resource settings.*	ID	To describe the steps and processes in the development and design of the mWellcare intervention.<br><br>**Qualitative method:** descriptive study	N/A<br><br>community setting	**Intervention: mWellcare Programme - mHealth-based electronic decision support (EDS) system**<br>Services including: **for EDS system:** (a) generate EDS recommendations for the management of diseases, (b) store electronic health records, (c) long-term monitoring and follow-up, (d) reminder message service for scheduled medication adherence and follow-up visits; **for training:** (e) centralized training on the clinical management guidelines to all physicians , (f) onsite training for NCD nurses in the management of hypertension, diabetes mellitus, depression, and tobacco and alcohol use, (g) training, onsite supervisor and support for nurses for orientation of the system.<br><br>**Control: none**	Lack of evidence-based, integrated, and systematic management of chronic conditions were major gaps identified. Experts in information technology, clinical fields, and public health professionals identified intervention components to address these gaps. Thereafter, clinical algorithm contextualized to primary care settings were prepared and the mWellcare intervention was developed. During the 2-month pilot, 631 patients diagnosed with hypertension and/ or diabetes were registered, with a follow-up rate of 36.2%. The major barrier was resistance to follow mWellcare recommended patient workflow, and to overcome it, we emphasized onsite training and orientation program to cover all health care team member in each CHC.
**Wang X, et al.(2018)**	*The Luohu Model: A Template for Integrated Urban Healthcare Systems in China*	CN	To introduce the Luohu model, to evaluate its effects and to explore lessons learned.<br><br>**Qualitative method:** descriptive study	N/A<br><br>ambulatory setting<br>community setting	**Intervention: The Reform of Integrated Service Delivery Network-Luohu Hospital Group (2015-2017)**<br>Summary: (a) system integration: global budget, 6 administrative centres, 6 resources-sharing centres; (b) organisational integration: close hospital group (including 5 hospitals, 1 research institute, 6 health resource sharing centres, 6 administrative centres, 23 CHCs); (c) professional integration: multidisciplinary family doctor teams, specialists set up studio in CHCs, integrated care for the elderly; (d) functional integration: platform for two-way referrals, health Luohu APP and 4G mobile nursing; (e) normative integration: shared vision in the hospital group, build trust between residents and family doctor teams.<br><br>**Control: none**	The Luohu hospital group consists of five district hospitals, 23 community health stations and an institute of precision medicine. The group adopted a series of professional, organizational, system, functional and normative strategies for integrated care, which was provided for the residents of Luohu, especially for the elderly population and patients with chronic conditions.<br><br>According to a preliminary evaluation of the past two years, the Luohu model showed improvement in the structure and process towards integrated care. New preventive programs conducted in the hospital group resulted in changes of disease incidence. Residents were more satisfied with the Luohu model. However, spending exceeded the global budget for health insurance because of short-term increases in the demand for health care.

**Nurjono M, et al.(2018)**	*Implementation of Integrated Care in Singapore: A Complex Adaptive System Perspective.*	SG	To analyse two integrated care initiatives - the Regional Health System (RHS) model and CARITAS Integrated Dementia Care in terms of characteristics and implications from complex adaptive system (CAS) perspective.<br><br>**Qualitative method:** descriptive study	N/A<br><br>inpatient setting<br>community setting<br>home setting	**Intervention: Regional Health System**<br>Summary: a network led by a major public hospital, working in close partnership with other healthcare providers (primary care providers, community hospitals, nursing homes, home care and day rehabilitation providers) and social care providers within the same geographical region.<br><br>**Intervention: CARITAS Integrated Dementia Care**<br>Services including: (a) regular case conference, (b) shared and transfer of care from hospital to primary and community care, (c) primary and community care providers trainings, (d) caregivers education and help-line .<br><br>**Control: none**	By considering the RHS and CARITAS as whole networks each comprising of interacting and adaptive components instead of separate entities within a bigger system, the CAS provided a new mind- set in surfacing issues associated to the implementation of these integrated care networks. In addition to important actors, systems, it informed understanding of relationships and dependencies between different parts of the network – revealing the lack of homogeneity, conformity and difficulties in designing any optimal system in advance given the many moving parts.
**Nurjono, M., et al.(2019)**	*Implementation fidelity of a strategy to integrate service delivery: learnings from a transitional care program for individuals with complex needs in Singapore.*	SG	To (i) evaluate the implementation fidelity of the NUHS-RHS TCP to explain the outcomes of the program, and (ii) inform further development of (similar) programs.<br><br>**Mixed methods:** realist evaluation study	42 Non-participatory observations, 44 medical records of patients and 2705 records (data analysis); 25 healthcare providers and 45 healthcare users (interviews)<br><br>inpatient setting<br>home setting	**Intervention: National University Health System-Regional Health System Transitional Care Programme**<br>Services including: (a) needs assessments, (b) development of personalized care plans, (c) care coordination (symptoms management, functional management, psychological support, medication management, home environment assessment, management of social issues, appointment management, promotion of self-care, referral to other services, advocacy and telephone monitoring), and (d) case closure (complete discharge or discharge to less or more intensive care).<br><br>**Control: none**	(a) low or moderate level of fidelity in most components (10 out of 14);<br>(b) the frequency or duration of the program components were varied based on the needs of users, availability of care coordinators (CC) and their confidence;<br>(c) variation in fidelity was influenced predominantly by: 1) complexity of the program, 2) extent of facilitation through guiding protocols, 3) facilitation of program implementation through CCs' level of training and confidence, 4) evolving healthcare participant responsiveness, and 5) the context of suboptimal capability among community providers

**Liang, L.L.(2019)**	*Impact of integrated healthcare: Taiwan's Family Doctor Plan.*	TW	To estimate the effects of shifting from usual to integrated care and locates contextual factors that may distort programme implementation.<br><br>**Quantitative method:** difference-in-difference design	168,043 patients in the panel database<br><br>ambulatory setting community setting	**Intervention: Family Doctor Plan**<br>Services including: (a) multidisciplinary teams, (b) case management: health assessment, patient education and creation of health profiles, and (c) integrated care pathways: referrals, follow-ups and feedbacks, and transitional care management, a 24-hour medical consultation telephone line, an operational plan for quality improvement.<br><br>**Control: programme eligible patients who had not assigned**	The programme reduced Continuity of Care Index (COCI) of provider continuity at the clinic level and continuity throughout the entire health care system, i.e. the clinic and hospital levels.<br><br>None of the incident rate ratios (IRRs) associated with the participation indicator was significant at the 5% level (95% confidence interval for the IRR: 0.92–1.00 for lipid profiles tests, 0.98–1.07 for renal function tests, 0.95–1.03 for liver function tests), showing that the FDP had no impact on test duplications.<br><br>The programme had no impact on avoidable admissions [95% confidence interval for the odds ratio (OR): 0.97– 1.19 for bacterial pneumonia, 0.87–1.07 for urinary tract infection], increased the odds of drug injection by 3.9% (95% confidence inter- val for the OR: 1.02–1.06) and increased the number of emergency department visits by 3.3% (95% confidence interval for the IRR: 1.02–1.05).
**Suriyawongpaisal, P., et al.(2019)**	*Assessing system-based trainings for primary care teams and quality-of-life of patients with multimorbidity in Thailand: patient and provider surveys.*	TL	To reveal the relationship between these systems-based trainings in diverse settings of primary care and quantified patient outcomes.<br><br>**Quantitative method:** cross-sectional survey	1,916 eligible patients across 8 selected provinces in Thiland participated<br><br>community setting	**Intervention: 12 Training Programmes for Primary Care Teams (n=1,916)**<br>Services including: **integrated primary care systems management:** (a) resource sharing, (b) community participation and inter-sectoral collaboration, (c) health information systems, (d) management skills of the leaders, (e) coordination and unity of teamwork, and (f) integrated service delivery.<br><br>**Control: none**	Health-related quality of life (HRQoL) were positively associated to the primary care teams (PCT) exposure to training programmes of District Health Management Learning (DHML) and Contracting Unit of Primary Care (CUP) Leadership Training Programmes.<br><br>Family Practice Learning (FPL) was negatively associated with patient's HRQoL.<br><br>The duration of time spent working within the PCT was also slightly negatively associated with patient's HRQoL.

**Ang, I.Y.H., et al.(2019)**	*Retrospective evaluation of healthcare utilisation and mortality of two post-discharge care programmes in Singapore.*	SG	To evaluate the impact on healthcare utilisation frequencies and charges, and mortality of a programme for frequent hospital utilisers and a programme for patients requiring high acuity post-discharge care as part of an integrated healthcare model.<br><br>**Quantitative method:** retrospective quasi-experimental study	1,648 eligible patients' records extracted through health administrated dataset<br><br>inpatient setting<br>home setting	**Intervention 1:National University Health System-Regional Health System Integrated Interventions and Care Extension programme (n=554)**<br>Services including: (a) case management for patients and caregivers, (b) follow-up home visits, (c) follow-up telephone calls, (d) community-based care management (home nursing, home hospice, outpatient clinic, social workers and corporate volunteers).<br><br>**Intervention 2: National University Health System-Regional Health System Transitional Care Programme (n=270)**<br>Services including: transitional care nurse-led care team: (a) medical support, (b) nursing support, (c) rehabilitative support and (d) psycho-social support, (e) community-based care management (home nursing, home hospice, outpatient clinic, social workers and corporate volunteers).<br><br>**Control: patients not enrolled in either programmes (n=554 for comparison of programme 1 and n=270 for programme 2)**	For programme 1, significantly higher all-cause inpatient admission charges (MR=2.38, 95%CI 1.44 to 3.92), emergency admission charges (MR=2.71, 95%CI 1.60 to 4.58) and emergency department attendance charges (MR=2.17, 95%CI 1.50 to 3.13) among intervention patients were observed when compared with the control patients. SOC attendance charges among intervention patients were not statistical different from the control patients (MR=1.31, 95% CI 0.96 to 1.79).<br><br>For programme 2, significantly higher post-enrolment emergency admission charges (MR=2.64, 95%CI 1.15 to 6.06) and post-enrolment SOC attendance charges (MR=4.50, 95%CI 2.82 to 7.18), among intervention patients were observed when compared with the control patients.

**Su D, et al.(2019)**	*Does capitation prepayment based Integrated County Healthcare Consortium affect inpatient distribution and benefits in Anhui Province, China? An interrupted time series analysis.*	CN	To compare the level and trend changes of inpatient and funds distribution, as well as inpatient benefits before and after the official operation of the capitation prepayment based Integrated County Healthcare Consortium (ICHC) in Anhui.<br><br>**Quantitative method:** a longitudinal and quasi-experimental study (pre and post analysis)	343,981 and 669,834 hospitalisation records of two counties extracted from National Dataset<br><br>ambulatory setting<br>inpatient setting	Intervention: The Reform of the Integrated County Healthcare Consortium<br>Summary: (a) integrated high-quality healthcare resources in the county, led by county- level hospitals (CHs), and jointly by township-level hospitals (THs) and village clinics and to establish a number of ICHCs that can provide integrated and continuous healthcare for residents within the ICHC, (b) formed a horizontal competition between different ICHCs, (c) established capitation prepayment of the the New Rural Cooperative Medical System (NRCMS) funds.<br><br>Control: none	The average hospitalisation expenses showed a decreasing trend and the actual compensation ratio increased significantly (p-value < 0.01).<br><br>Most of the indicators in the two counties performed well, and the effect of such policy was better in Funan County than in Dingyuan County.<br><br>The distribution of inpatients and NRCMS funds outside the county after the reform in Dingyuan showed an increasing trend (0.27, 95%CI: 0.12 to 0.42, p-value < 0.01; 0.70, 95%CI: 0.32 to 1.09, p-value < 0.01) and the distribution of inpatients and NRCMS funds in THs showed a more obvious upward trend after the reform in Funan (0.44, 95%CI: 0.22 to 0.67, p-value < 0.001; 0.34, 95%CI: 0.23 to 0.45, p-value < 0.001).

**Liang D, et al.(2020)**	*Building a People-Centred Integrated Care Model in Urban China: A Qualitative Study of the Health Reform in Luohu*	CN	To examine the policy-making and implementation processes of the health reform in Luohu, including its policy environment and health care market context, the actors involved in the reform and their roles, and how the reform strategies were implemented to achieve integrated care.<br><br>**Qualitative method:** semi-structured interviews and focus groups	36 informants, with government officials were purposely recruited and health workers were randomly selected<br><br>ambulatory setting community setting	**Intervention: The Reform of Integrated Service Delivery Network-Luohu Hospital Group (2015-2019)**<br>Summary: (a) system integration: integrated health care with public health services (e.g., health education, free vaccination, etc.) and social services (e.g., home care for disabled elders, community care in collaboration with daycare centers, etc.); (b) organisational integration: merged resources of 5 district-level public hospitals and 23 public community health centers (CHCs), single legal entity (Luohu Hospital Group), 6 administrative centres and 6 supporting centres; (c) professional integration: motivated specialists to train providers and work part-time at CHCs, consolidated professional resources across hospitals; (d) clincial integration: a formal two-way referral system, timely decision support from specialists at hospitals to primary care doctors , encouraged providers to integrate clinical pathways; (e) functional integration: shared key support functions including shared information system; (f) normative integration: guided by a "health-centered" perspective and shared the goals of "less illness, fewer hospitalisations, less burden, and better care".<br><br>**Control: none**	The reform in Luohu took place in a competitive health care market, based on the comprehensive health reform in Shenzhen. Under the strong leadership of the district government, the reform adopted comprehensive strategies to strengthen primary care and care coordination, improve the quality and efficiency of health care delivery, and promote population health. The reform achieved a high level of organisational integration but was still in the process of fulfilling professional and clinical integration.

**Leung TH, et al.(2020)**	*The effectiveness of an emergency physician-led frailty unit for the living-alone elderly: A pilot retrospective cohort study.*	HK	To assess the effectiveness of frailty-care model in this locality.<br><br>**Quantitative method:** retrospective cohort study	190 eligible patients were recruited from Emergency Medicine Ward (EMW) of the hospital<br><br>inpatient setting community setting	**Intervention: emergency physician–led frailty-care unit (n=150)**<br>Services including: (a) a mandatory and complete input of electronic Patient Assessment Form (PAF) by nursing staff, (b) a comprehensive enquiry of social background (e.g. safety bell, financial support and attendance to day care centre) (c) a mandatory bundled referral to the pre-discharge nursing service team, physiotherapist and occupational therapist upon admission, (d) daily ward round by a consultant and/or specialist emergency physician with nursing and allied health team to decide on a management plan, (e) a target of discharge within 72 h of admission.<br><br>**Control: traditional general care (n=40)**<br>Services including: (a) an optional or partial input of electronic Patient Assessment Form (PAF) by nursing staff, (b) a basic enquiry of social background would be conducted (e.g. activity of daily living and main carer) (c) referral to relevant nursing and allied health team individually as deemed necessary by the duty doctor, (d) daily ward round by a specialist or higher trainee in emergency medicine.	The length of stay in the acute hospital is significantly shortened (2.38 vs 3.27days, p=0.00018) than in the general group.<br><br>The transferral rate to a convalescent hospital was less in frailty group (21.3% vs 42.5%, p=0.00655).<br><br>There was no significant difference in terms of total (7.10 vs 10.99days, p=0.09638) and convalescent (22.09 vs 18.16days, p=0.48183) length of stay between frailty group and general group, respectively.<br><br>The transferral rate to convalescent hospital was less in the frailty group (21.3% vs 42.5%, p = 0.00655).<br><br>The 28-day re-attendance rate was similar (26.7% vs 15.0%, p = 0.12589).

**Mitsutake S, et al.(2020)**	*Associations of Hospital Discharge Services With Potentially Avoidable Readmissions Within 30 Days Among Older Adults After Rehabilitation in Acute Care Hospitals in Tokyo, Japan.*	JP	To examine the associations of major discharge services covered under health insurance (discharge planning, rehabilitation discharge instruction, and coordination with community care) with 30-day PAR among older adults who received rehabilitation while admitted at acute care hospitals in Tokyo, Japan.<br><br>**Quantitative method:** retrospective cohort study	31,247 eligible patients' medical records were obtained from the medical claims database<br><br>inpatient setting<br>community setting	**Intervention: discharge services (n=31,247)**<br>Services including: (a) discharge planning , (b) rehabilitation discharge instruction to educate patients and their families on self-management in daily living, (c) instructions for community care at discharge to provide post discharge instructions (eg. medication safety at home, shared information with community healthcare workers), (d) coordination with long-term care (case needs assessment and long-term care plan based on medical, physical, or environmental problems).<br><br>**Control: none**	The discharge services were not significantly associated with 30-day PAR. The odds ratios were 0.962 (95% confidence interval [CI], 0.805-1.151) for discharge planning, 1.060 (95% CI, 0.916-1.227) for rehabilitation discharge instruction, and 1.118 (95% CI, 0.817-1.529) for coordination with community care.<br><br>In contrast, the odds of 30-day PAR among patients with home medical care services were 1.431 times higher than those of patients without these services (P<.001).<br><br>The odds of 30-day PAR among patients with a higher number (median or higher) of rehabilitation units were 2.031 times higher than those of patients with a lower number (below median) (P<.001).<br><br>Also, the odds of 30-day PAR among patients with a higher Hospital Frailty Risk Score (median or higher) were 1.252 times higher than those of patients with a lower score (below median) (PZ.001).
Nurjono M, et al.(2020)	*Shifting care from hospital to community, a strategy to integrate care in Singapore: process evaluation of implementation fidelity.*	SG	To conduct process evaluation to examine the implementation fidelity of the National University Health System (NUHS) Regional Health System (RHS) program at providers, organizational, and system levels.<br><br>**Mixed methods:** realist evaluation study	11 non-participatory patients were enrolled through convenient sampling, 30 eligible patient's medical records were randomly enrolled, with 29 analysed, 25 healthcare providers involved in the program were interviewed<br><br>ambulatory setting<br>community setting	**Intervention: Right-Site Care programme**<br>Service including: (a) referral of suitable patients by specialists at Specialist Outpatient Clinics (SOCs), (b) coordination of care under one dedicated physician, (c) stratification of care and care location according to the patients' need and preference, (d) preparation of transition from SOCs to other appropriate sites (patients education, appointment management, provision of referral/memo), (e) support for primary and community care providers by a team of hospital-based providers, (f) multi-disciplinary case conference to refine care plans.<br><br>**Control: none**	Four out of six programme components were implemented with low level of fidelity, and 9112 suitable patients were referred to the program while 3032 (33.3%) declined to be enrolled. Moderating factors found to influence fidelity included: (i) complexity of program, (ii) evolving providers' responsiveness, (iii) facilitation through synergistic partnership, training of PCC providers by specialists and supportive structures: care coordinators, guiding protocols, shared electronic medical record and shared pharmacy, (iv) lack of organization reinforcement, and (v) mismatch between program goals, healthcare financing and providers' reimbursement.

Abbreviations: JP=Japan, HK=Hong Kong, TL=Thailand, CH=China, SG=Singapore, TW=Taiwan, ID=India, N/A=not applicable.

Appendix 5. Summary of IC programmes

Table S4. Summary of number of IC programmes for each country by IC component and element

Components/elements	SG	No. of programmes	HK	No. of programmes	CN	No. of programmes	JP	No. of programmes	ID	No. of programmes	TW	No. of programmes	TL	No. of programmes	Total
**<u>Multimorbidity</u>**	**6**	**9**	**2**	**5**	**0**	**3**	**0**	**3**	**1**	**1**	**1**	**1**	**1**	**1**	**11**
**<u>Environment</u>**															
Welfare services	1	SGH-TC	2	CM, EPFU			1	VP							4
Housing	2	SGH-TC (home care equipment, home safety), TC (home environment assessment)	2	HSTCM (housing assistance), EPFU	1	Luohu (home assessment)									5
Social network			1	CM(family)											1
Transport															0
Financing	1	THC-IPU	1	CM											2
Community	4	ACTION, SGH-THC, SGH-TC, NICE (community services assessment or activation to support patients' care, coordination of referral within community)	2	CM(supportive groups), TCM(service environment), HSTCM			3	VP, ICCS (livelihood support services), HDS (instruction regarding community care)							9
**<u>Service delivery</u>**															
Micro level															
Person-centred	6	ACTION, SGH-THC, SGH-TC, TC, NICE, THC-IPU	5	CM, TCM, NCM, HSTCM, EPFU			3	VP, ICCS, HDS	1	mWellcare	1	FDP(case management)			16

Tailored	7	ACTION, SGH-THC, TC, NICE, RSC, AMU, THC-IPU	4	CM, TCM, HSTCM, EPFU			3	VP(case conference), ICCS(case conference), HDS	1	mWellcare	1	FDP(case management)			16
Self-management	4	ACTION, SGH-THC, TC, THC-IPU	3	CM, TCM, HSTCM			2	VP, HDS	1	mWellcare	1	FDP(patient education)			11
Pro-active	7	ACTION, SGH-THC, SGH-TC, TC, NICE, RSC, THC-IPU	5	CM, TCM, NCM, HSTCM, EPFU	2	JHC(screening), ICHC(health education, promotion)	2	VP, HDS	1	mWellcare					17
Informal caregiver involvement	4	SGH-THC, SGH-TCP, NICE, THC-IPU	1	CM			2	VP(emotional support), HDS							7
Treatment interaction	3	SGH-THC, TCP, THC-IPU					1	HDS(medication safety)							4
Continuity	7	ACTION, SGH-THC, SGH-TC, TCP, NICE, RSC, THC-IPU	3	CM, TCM, EPFU			2	VP(coordination of services), HDS(coordination with LTC+community)	1	mWellcare(nurse and physician)			1	PCT(teamwork, resource sharing)	14
Meso level															
Organisational & structural integration	2	RHS, THC-IPU(physical locality)	2	TCM(multipurpose day centre), EPFU	3	JHC, Luohu, ICHC									7
Continuous quality improvement system					1	Luohu(quality management centre)									1
Macro level															
Market regulation															
Polices to integrate care across organisations & sectors	1	RHS			3	JHC, Luohu, ICHC	1	HDS							5
Service availability & access	1	RHS			3	JHC, Luohu, ICHC									4

**<u>Leadership & governance</u>**															
Micro level															
Shared decision-making	7	ACTION, SGH-THC, SGH-TC, TCP, NICE, RSC, THC-IPU	4	CM, TCM, HSTCM(mutual goal with patients), EPFU(multidisciplinary approach)	1	Luohu(GP&physician)	1	HDS(hosptail+community/LTC workers)	1	mWellcare(decision-support system)			1	PCT(teamwork)	15
Individualised care planning	7	ACTION, SGH-THC, SGH-TC, TC, NICE, RSC, THC-IPU	3	CM, TCM, EPFU	1	Luohu(GP&physician)	2	ICCS(discharge planning), HDS	1	mWellcare(decision-support system)	1	FDP(patient profile)			15
Coordination tailored to complexity	7	ACTION, SGH-THC, SGH-TC, TC, NICE, RSC, THC-IPU	5	CM, TCM, NCM, HSTCM, EPFU			2	VP, HDS	1	mWellcare(decision-support system)	1	FDP(patient profile)			16
Meso level															
Supportive leadership	8	ACTION, SGH-THC, SGH-TC, TC, RHS, NICE, RSC, THC-IPU	2	CM, TCM(trust)	2	JHC, Luohu							1	PCT(inter-sectoral collaboration)	13
Clear accountability	1	THC-IPU	2	CM, HSTCM	1	Luohu(shared community of accountability)									4
Performance-based management	3	TC, RHS, THC-IPU			3	JHC, Luohu, ICHC(performance assessment)									6
Culture of shared vision, ambition, values	2	RHS, THC-IPU(due to same physical locality)	1	TCM(NCM-client relationship)	1	Luohu(due to affiliation)									4
Macro level															

Policy & action plans on chronic diseases & multimorbidity	5	TC, RHS, NICE, RSC, THC-IPU			3	JHC, Luohu, ICHC									8
Political commitment	5	TC, RHS, NICE, RSC, THC-IPU			2	JHC, Luohu, ICHC									7
**<u>Workforce</u>**															
Micro level															
Multi-disciplinary team	1	TC, SGH-THC	3	CM, TCM, EPFU			1	ICCS(multidisciplinary service network)							5
Named coordinator	7	ACTION, SGH-THC, SGH-TC, TC, NICE, RSC, THC-IPU	4	CM, TCM, NCM, HSTCM											11
Core group	7	ACTION (case transition team), SGH-THC, SGH-TC, TC, NICE (care manager team), RSC, THC-IPU	4	CM, TCM, HSTCMP, EPFU	1	Luohu(family doctor team)					1	FDP(community health care group)			13
Meso level															
Continuous [professional] development	2	SGH-THCP(appropriate use of time and resources, coordinating care with specialies and multidisciplinary team members), AMU (management skills)	1	TCM(stress, NCM-client relationship)					1	mWellcare	1	FDP(management skills)	1	PCT(skills of self-assessment)	6
Informal caregiver support	4	SGH-THC, SGH-TC, NICE, THC-IPU					2	VP(emotional support), HDS							6

New professional roles	1	AMU			1	Luohu(family doctor team)			1	mWellcare(shift task from doctors)	1	FDP	1	PCT(task shifting to primary setting)	5
Macro level															
Workforce-demography match	1	RHS			3	JHC (NCD), Luohu(screening for diseases with high morbidity and mortality), ICHC(NCD)					1	FDP(chonic&non-chronic)	1		6
Educational & workforce planning					2	JHC(mentorship system), Luohu(recruitment)									2
**<u>Financing</u>**															
Micro level															
Coverage & reimbursement	2	ACTION (no cost), TC(high subsidies)			2	Luohu (social insurance), ICHC(insurance)	1	ICCS(LTCI)							5
Out of pocket costs	2	SGH-THC, THC-IPU													2
Financial incentives					1	Luohu (reforming health insurance payment, increasing funding, adjust prices)					1	FDP(provider incentives: case management fees, performance bonuses and a shared-saving model)			2
Meso level															
Incentives to collaborate					2	Luohu (global budget, surplus as staff bonuses), ICHC(pay for diseases)									2

Risk adjustment							1	HDS(diagnosis procedure combination system)			1	FDP (shared-savings model)			2
Shared savings					2	Luohu (global budget, surplus as staff bonuses), ICHC(capitation prepayment, balance holds for use)					1	FDP(global budget)			3
Secured budget					1	ICHC(prepayment)									1
Business case															0
Macro level															
Financial system for health- & social care					1	Luohu(funding mechanism for hospital and CHC)									1
Stimulating investments in innovative care models	1	TC (funding)			1	Luohu(salary reform)									2
Equity & access															0
**Technologies & medical products**															
Micro level															
EMRs & patient portals	4	SGH-THC, TC, RSC, THC-IPU	3	CM, NCM, EPFU	1	Luohu			1	mWellcare					9
E-health tools					1	Luohu(app to improve literacy)	1	VP(videophone)	1	mWellcare					3
Assistive technologies							1	VP(videophone)	1	mWellcare					2
Remote monitoring							1	VP(videophone)	1	mWellcare					2
Meso level															
Shared information systems	2	TC, THC-IPU	1	CM	2	JHC(appointment platform), Luohu(imaging sharing, EHR)			1	mWellcare					6

Interoperable systems	2	NICE, RSC(shared hospital pharmacy)			2	JHC(referral platform), Luohu(referral system)									4
Macro level															
Policies fostering technological innovation															0
Access to technologies & medical products															0
**<u>Information & research</u>**															
Micro level															
Individual level data	6	ACTION, SGH-THC, TC, NICE, RSC, THC-IPU	1	CM					1	mWellcare					8
Individual risk prediction	1	AMU													1
Meso level															
Data ownership & protection							1	VP							1
Innovative research [methods]															0
Risk stratification							1	HDS(diagnosis procedure combination system)							1
Macro level															
Privacy & data protection legislation															0
Policies that stimulate research in integrated care & multimorbidity															0
Access to information															0
**<u>Monitoring</u>**															

Micro level															
Monitoring of changes between face to face encounters	2	SGH-TC, THC-IPU(home visit)													2
Monitoring of care plans	5	ACTION(telephone+home visit), SGH-THC(telephone), SGH-TC(telephone), NICE(home visit+telephpne), THC-IPU(home visit)	4	CM(database, home visit, telephone), TCM (telephone), NCM, HSTCM(telephone)			1	VP	1	mWellcare(SMS)					11
Monitoring of self-management			2	NCM, HSTCM											2
Monitoring of clinical indicators			1	CM					1	mWellcare					2
Monitoring of preferences															0
Meso level															
Quality improvement system					1	Luohu(quality control in admin centre)			1	FDP(operational plan)					2
Macro level															
Support workforce-demography match	1	RHS													1
Provide epidemiological data															0

Abbreviations: JP=Japan, HK=Hong Kong, TL=Thailand, CN=China, SG=Singapore, TW=Taiwan, ID=India, SI=system integration, OI=organisational integration, PI=professional integration, CI=clinical integration, FI=functional integration, NI=normative integration, HI=horizontal integration, VI=vertical integration, MM=multimorbidity, N/R=Not reported, HHSPV=Home Healthcare Services Provided with Videophones, CM=Case Management, TCM=Targeted Case Management, FDP=Family Doctor Plan, TPPCT=Training Programmes for Primary Care Teams, ACTION=The Aged Care Transition, SGH-THC=Singapore General Hospital transitional home care, ICCS=Integrated Community Care System, NCM=Nurse-led Case Management Programme, HSTCM=Health-Social Transitional Care Management, SGH-TC=Singapore General Hospital Transitional Care, NUHS-RHS TC =National University Health System-Regional Health System Transitional Care, RHS=Regional Health System, JHC=Joint Health Centre, HDS=Hospital Discharge Services, NUHS-RHS NICE=National University Health System-Regional Health System Integrated Interventions and Care Extension, RSC=Right-Site Care, AMU=Acute Medical Unit, THC-IPU=Transitional Home Care-Integrated Practice Units, ICHC=Integrated County Healthcare Consortium, EPFU=Emergency Physician–led Frailty-care Unit.

Appendix 6. Number of IC programmes categorised by elements and components

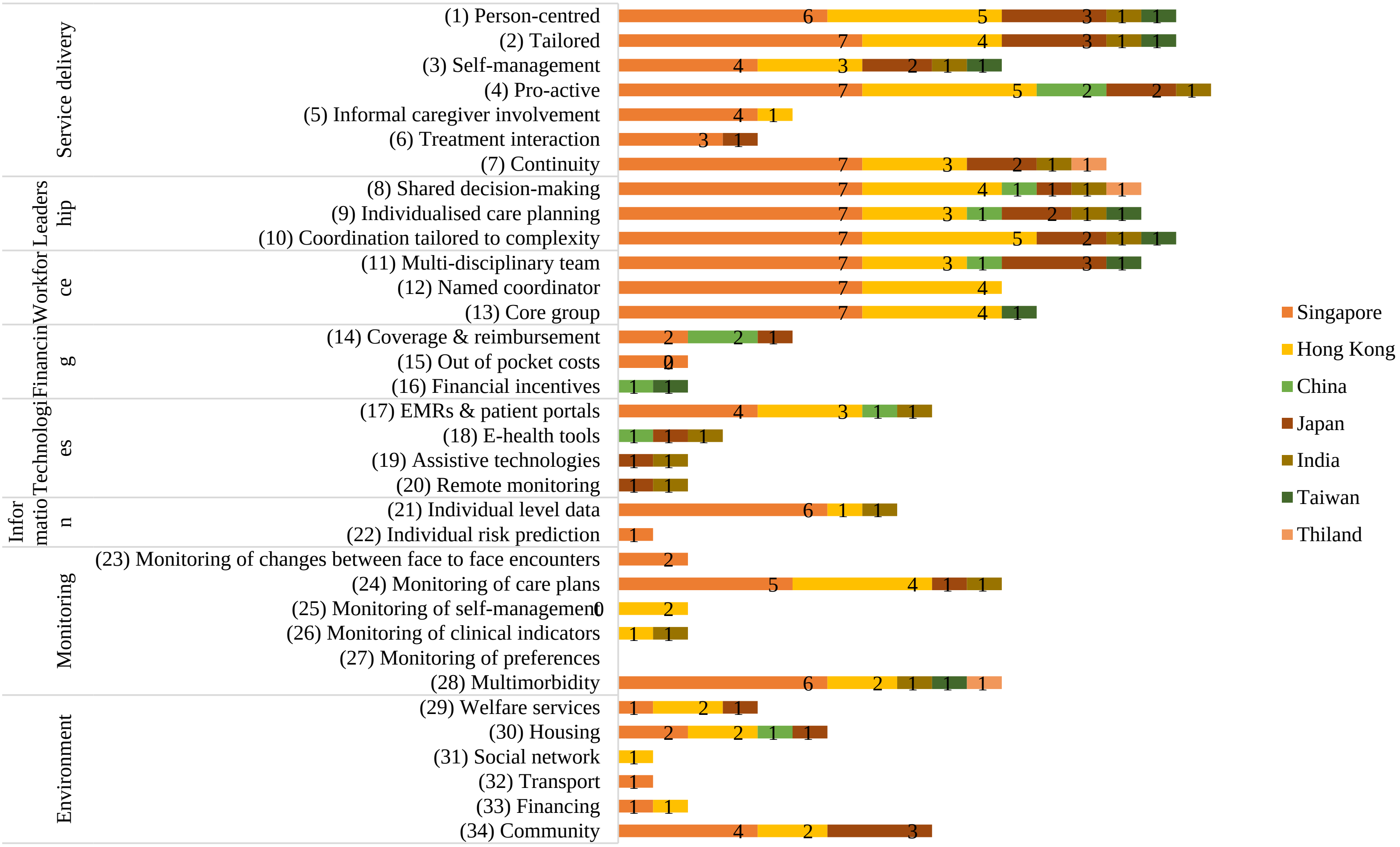

Fig S1. Number of IC programmes reporting micro-level elements under each component

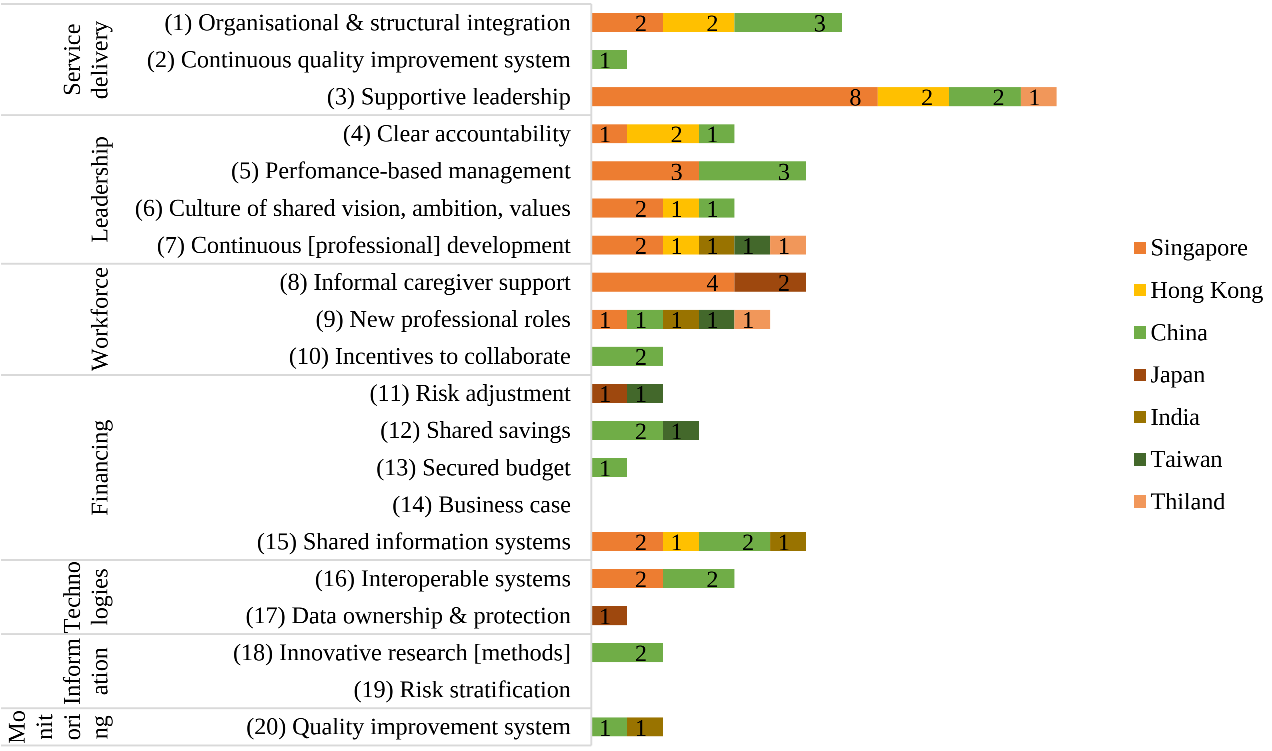

Fig S2. Number of IC programmes reporting meso-level elements under each component

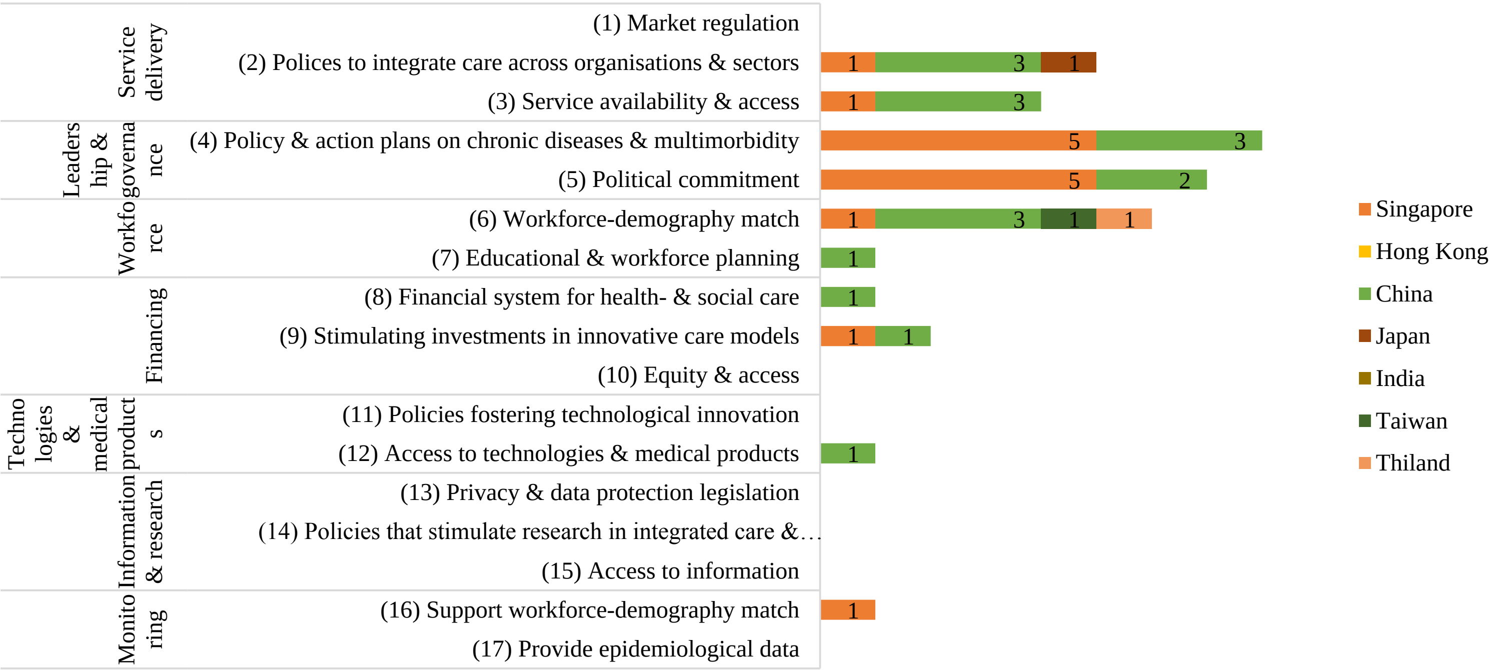

Fig S3. Number of IC programmes reporting macro-level elements under each component
